# Natural Products Impacting DNA Methyltransferases and Histone Deacetylases

**DOI:** 10.3389/fphar.2020.00992

**Published:** 2020-08-13

**Authors:** Sergi Herve Akone, Fidele Ntie-Kang, Fabian Stuhldreier, Monique Bassomo Ewonkem, Alexandre Mboene Noah, Simon Eitel Misse Mouelle, Rolf Müller

**Affiliations:** ^1^Department of Chemistry, Faculty of Science, University of Douala, Douala, Cameroon; ^2^Department of Microbial Natural Products, Helmholtz Institute for Pharmaceutical Research Saarland (HIPS), Helmholtz Centre for Infection Research and Department of Pharmacy, Saarland University, Saarbrücken, Germany; ^3^Department of Chemistry, Faculty of Science, University of Buea, Buea, Cameroon; ^4^Institute for Pharmacy, Martin-Luther-Universität Halle-Wittenberg, Halle (Saale), Germany; ^5^Institut für Botanik, Technische Universität Dresden, Dresden, Germany; ^6^Medical Faculty, Institute of Molecular Medicine I, Heinrich Heine University Düsseldorf, Düsseldorf, Germany; ^7^Department of Biochemistry, Faculty of Science, University of Douala, Douala, Cameroon

**Keywords:** epigenetics, natural products, inhibition, DNA methyltransferases, histone deacetylases, cancer

## Abstract

Epigenetics refers to heritable changes in gene expression and chromatin structure without change in a DNA sequence. Several epigenetic modifications and respective regulators have been reported. These include DNA methylation, chromatin remodeling, histone post-translational modifications, and non-coding RNAs. Emerging evidence has revealed that epigenetic dysregulations are involved in a wide range of diseases including cancers. Therefore, the reversible nature of epigenetic modifications concerning activation or inhibition of enzymes involved could be promising targets and useful tools for the elucidation of cellular and biological phenomena. In this review, emphasis is laid on natural products that inhibit DNA methyltransferases (DNMTs) and histone deacetylases (HDACs) making them promising candidates for the development of lead structures for anticancer-drugs targeting epigenetic modifications. However, most of the natural products targeting HDAC and/or DNMT lack isoform selectivity, which is important for determining their potential use as therapeutic agents. Nevertheless, the structures presented in this review offer the well-founded basis that screening and chemical modifications of natural products will in future provide not only leads to the identification of more specific inhibitors with fewer side effects, but also important features for the elucidation of HDAC and DNMT function with respect to cancer treatment.

## Introduction

Natural products originating from diverse sources including plants, microorganisms, and marine sponges are capable to influence epigenetic modifications (Deng et al., 2018; Lascano et al., 2018). Epigenetics refers to heritable changes in gene expression as well as chromatin structure without change in a DNA sequence (Dawson, 2017). Enzymes involved in these modifications were already identified and include DNA methyltransferases, histones acetyltransferases/deacetylases, histone lysine as well as arginine methyltransferases and histone demethylases (Deng et al., 2018). The most common epigenetic modifications include DNA methylation, chromatin remodeling, histone post-translational modifications, and silencing of gene expression through non-coding RNAs (Allis and Jenuwein, 2016). Epigenetic dysregulations have been shown to be involved in several diseases such as cancers, neurodegenerative, or parasitic diseases and obesity (Duraisingh and Skillman, 2018; Ferioli et al., 2019). Cancer is a disease strongly tied to epigenetic changes which lead to silencing of tumor suppressor genes and thus promote tumor formation and proliferation (Jones and Baylin, 2007; Esteller, 2008). Therefore, due to the reversible nature of epigenetic modifications with respect to activation or inhibition, the genes and proteins representing key epigenetic players are considered as prime targets for the treatment and prevention of cancers (Ellis et al., 2009). In this review, we focus on natural products from plants, sponges, bacteria, and fungi that may serve as leads for drug discovery and possible further development for the treatment of cancers. We describe natural compounds of all biosynthetic classes inhibiting enzymes involved in the major epigenetic modifications regulating gene expression which include DNA methyltransferases (DNMTs) and histone deacetylases (HDACs). Emphasis will be on their biological properties and mode of action. Most of the natural products seem to display an indirect effect on HDACs and DNMTs and we aim to provide in this review all the data reported in the literature. It is worth to mention that currently, modifiers of DNMTs and HDACs are the only two classes of epigenetic drugs under investigation in the clinical setting.

## DNA Methyltransferases Inhibitors (**Figure 1**)

The transfer of methyl groups to DNA is performed by DNA methyltransferases (DNMTs). These enzymes create 5-methylcytosines (5mCs) which lead to gene repression. The development of novel drugs targeting cancer and other diseases involve DNMTs as epigenetic targets (Medina-Franco et al., 2015). DNMT1, DNMT3a, and DNMT3b were identified as the three catalytically active DNMTs in mammals. DNMT1 is described as the maintenance methyltransferase, while DNMT3a and DNMT3b are *de novo* methyltransferases (Auclair and Weber, 2012). A crucial epigenetic modification is the modulation of the activity of DNA methyltransferases which affect DNA repair mechanisms in the cells or gene expression. The pathogenesis of human cancer is partly due to aberrant modiﬁcations in the activity of DNMTs (Jasek et al., 2019). DNMTs modulate DNA methylation and in general perform methylation of nitrogen (*N*-methylation), oxygen (*O*-methylation), and carbon (*C*-methylation). Those events are universal processes critical to all organisms. The *O*-methylation patterns of polyhydroxylated small molecules in plants, are essential using the same or similar intermediates and substrates to generate final product distribution through multiple branched biosynthetic pathways (Okano et al., 1999; Zubieta et al., 2001). In any organism, methylation is essential in the management of normal biological activities (Esteller, 2007). Methylation can cause direct suppression of gene expression even though it is an heritable change in the DNA without a modification of the sequence (Das and Singal, 2004). Hypomethylation and hypermethylation of DNA are seen in cancer cells (Qi and Xiong, 2018). The expression of pro-metastatic genes and quiescent proto-oncogenes is assisted by hypomethylation, and thus increase the progression of tumors. Silencing of genes influencing important cellular signaling pathways can be due to hypermethylation of the promoter regions of tumor-suppressor genes which play an important role in neoplastic transformation of cells (Qi and Xiong, 2018).

**Figure 1:**
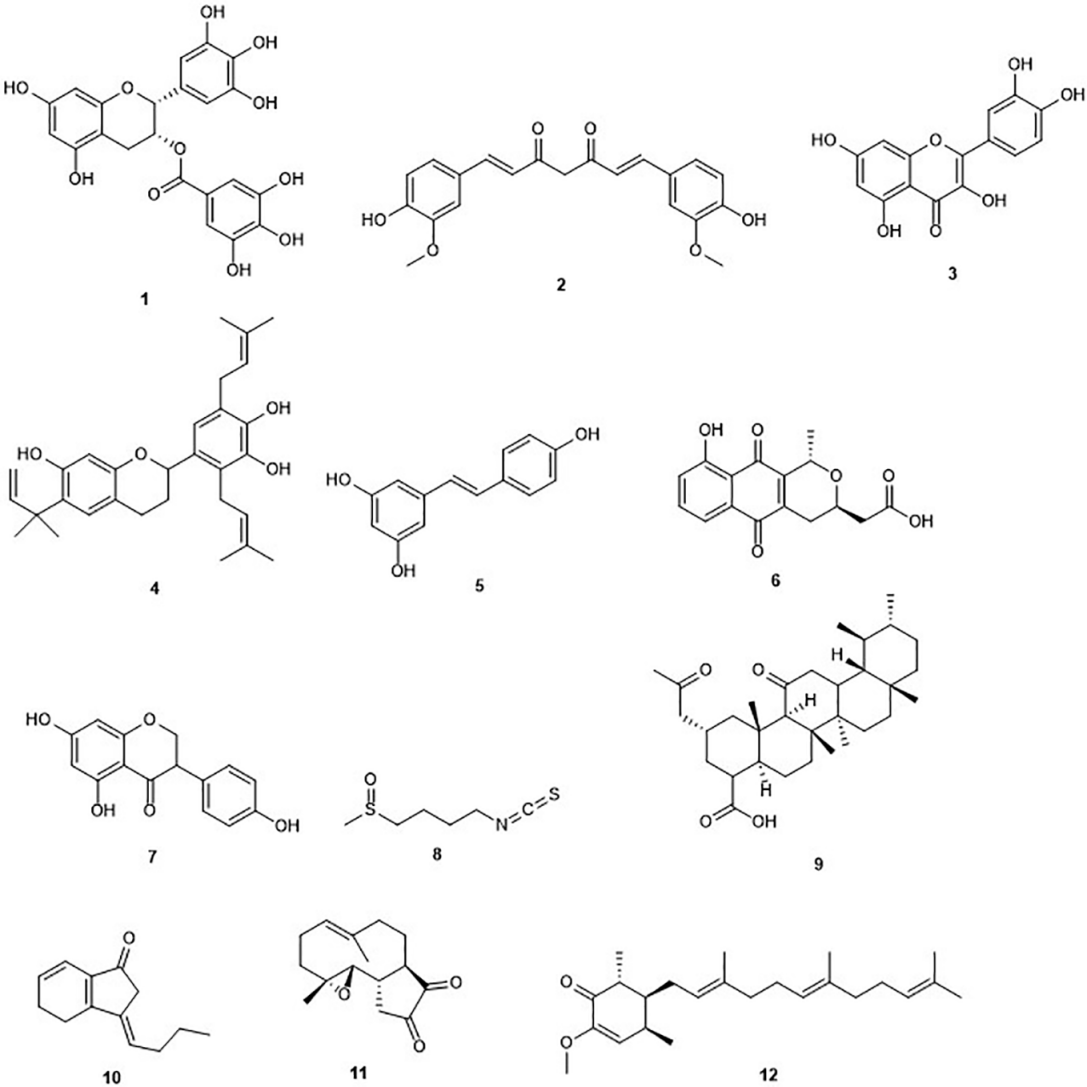
Structures of DNA methyltransferases inhibitors.

There are few drugs targeting DNA methyltransferase as inhibitors (DNMTIs), such as azacytidine and decitabine which are the most outstanding epigenetic drugs extensively utilized as epigenetic modulators. However, their application for oncological diseases is restricted by their relative toxicity and poor chemical stability (Gnyszka et al., 2013). Promising results in cancer treatment *via* influencing DNA methylation are expected from natural products isolated from plant, animals, and microorganisms. Indeed, bioactive phytochemicals that are widely available and exhibit less systemic toxicity have shown signiﬁcant anticancer properties. The potential utilization of natural products in cancer chemoprevention and/or therapy and their plausible role as epigenetic modulators have been diligently evaluated (Uramova et al., 2018). Moreover, diverse epigenetic modifications such as DNA methylation patterns including the global hypomethylation of oncogenes and the hypermethylation of tumor-suppressor genes are influenced by natural products from diverse origins. Nature is thus, a promising source of DNMTIs that may be useful in the treatment of cancer (**Table 1**). However, confirmation of the potential advantageous epigenetic effects after extended utilization in humans is highly dependent on successful delivery to allow for effective concentrations at the target cells and requires well-controlled clinical studies (Jasek et al., 2019). Moreover, most of the natural products target all DNMT isoforms leading to a low specificity that might limit their potential use in clinical development.

**Table 1:** Selected DNMT inhibitors.

Compound name	IC_50_ Values	DNMTIsoforms	Reference
(-)-epigallocatechin-3-gallate	0.47 µM	DNMT1	Lee et al., 2005
Curcumin	0.30 µM	DNMT1	Kwon et al., 1998
Kazinol Q	7.00 µM	DNMT1	Weng et al., 2014
Nanaomycin A	0.50 µM	DNMT3b	Kuck et al., 2010
Parthenolide	3.50 µM	DNMT1	Liu et al., 2009a
Antroquinonol D	5.00 µM	DNMT1	Wang et al., 2014

### (–)-Epigallocatechin-3-gallate

Green tea contains (–)-epigallocatechin-3-gallate (EGCG) (**1**) **Figure 1**) as the profuse catechin (Cabrera et al., 2006). Green tea is a popular natural beverage worldwide known to possess cancer preventive activities due to the presence of polyphenols such as catechins (Yang and Wang, 2010). EGCG and green tea polyphenols have demonstrated interesting chemopreventive effects and probable cancer chemotherapeutic effects against diverse cancers including liver, stomach, breast, lung, and skin (Nihal et al., 2005; Zhou et al., 2013; Khan et al., 2014). EGCG was first reported in 2003 to inhibit DNMT activity with an IC_50_ value of 20 µM and reactivate methylation-silenced genes in cancer cells (Fang et al., 2003). The same study showed that EGCG non-covalently binds to the DNMT1 catalytic active site. Another study reported that EGCG was a more potent inhibitor of DNA methyltransferase, compared to other polyphenols, with IC_50_ values between 0.21 and 0.47 µM in a direct inhibition assay (Lee et al., 2005). The authors demonstrated that the inhibitory interaction with the catalytic site of human DNMT1, and the high-affinity is due to the gallic acid moiety of EGCG (Lee et al., 2005). Moreover, its binding with the enzyme is stabilized by Mg^2+^ (Lee et al., 2005). Hussain and co-workers reported that EGCG inhibits the growth of HeLa cancer cells in a dose- and time-dependent manner through the induction of apoptosis (Sharma et al., 2012). Later on, it was found that EGCG inhibits DNA methyltransferases in HeLa cells in a time-dependent manner by reversal expression of diverse tumor-suppressor genes (Khan et al., 2015). Indeed, treatment of HeLa cells with EGCG, displayed a reduced expression of DNMT3b and docking studies indicated a direct binding of EGCG in the substrate-binding pocket of this enzyme. Another study also highlighted the potential of EGCG to inhibit DNMT in a breast cancer cell line (Meeran et al., 2011). However, Medina-Franco and co-workers contradicted the previous results suggesting a negligible inhibitory activity of (−)-epigallocathechin-3-gallate (EGCG) and curcumin (Medina-Franco et al., 2011).

### Curcumin

The polyphenol curcumin (**2**) has been isolated from *Curcuma longa* and is a major yellow pigment extracted from the popular Indian spice turmeric (Peng et al., 2017; Soleimani et al., 2018). It has been reported in the treatment of skin wounds, certain tumors, as well as inflammation (Maheshwari et al., 2006). However, curcumin is known as a PAINS (pan-assay interference compounds) and thus its bioactivities have to be analyzed with care (Nelson et al., 2017). A recent report indicated an inhibition of the growth of myeloma cells by curcumin with an IC_50_ value of 10 µM (Chen et al., 2019a). Curcumin has been reported to induce cell cycle arrest at G1/S phase in androgen-sensitive prostate cancer LNCaP cells and androgen insensitive PC-3 cells (Srivastava et al., 2007). Curcumin was reported to covalently block the catalytic thiolate of DNMT1 with an IC_50_ value of 30 nM after 72 h of treatment and leading to an inhibitory effect on DNA methylation (Liu et al., 2009b). Yu et al. (2013), showed a down-regulation in DNMT1 expression caused by curcumin in acute myeloid leukemia (AML) cell lines, both *in vitro* and *in vivo* (Yu et al., 2013). The same study highlighted reduction in the expression of positive regulators of DNMT1, Sp1 and p65 by curcumin. In AML cell lines, the latter results correlated with a decrease in binding of these transcription factors to the DNMT1 promoter. Curcumin displayed an inhibitory effect on DNMT in three colorectal cells lines including CT116, HT29 and RKO (Link et al., 2013). The DNA methylation changes in the same study occurred only in a subset of primarily partially methylated genes, and in a time-dependent manner. As mentioned earlier, the DNMT inhibition activity of curcumin has also been questioned (Medina-Franco et al., 2011).

### Quercetin

Quercetin (**3**), an ubiquitous dietary flavonoid, is commonly found in fruits, vegetables, and beverages. It has attracted considerable attention owing to its potent antioxidant and antiproliferative activities. The induction of apoptosis and cell cycle arrest by quercetin in several cancer cell lines among which breast carcinoma, human esophageal squamous cell carcinoma, and prostate cancer cell lines has been demonstrated (Nair et al., 2004; Jeong et al., 2009; Zhang et al., 2009). Recently, it was reported that the pro-apoptotic effect of quercetin was mediated by inhibition of DNMT, especially DNMT1 and DNMT3a *in vitro* and human xenograft models (Alvarez et al., 2018). Another study displayed a concentration-dependent effect on hypermethylation of the tumor suppressor gene, p16^INK4a^ when the human colon carcinoma RKO cells were treated by quercetin (Tan et al., 2009). This led to the reversal of the abnormal hypermethylation status of this gene after 120 h exposure to quercetin. Quercetin is currently undergoing phase I clinical trials in combination with green tea extracts (Zwergel et al., 2016). However, in the last update at ClinicalTrial.gov, the status is “recruiting” and no results have been published yet. Furthermore, it was reported that quercetin could be used in the treatment of diseases such as diabetes, in which its methylglyoxal (MGO) adduct plays an important function (van den Eynde et al., 2018).

### Kazinol Q

Kazinol Q (**4**) is a natural flavan isolated from the root of *Broussonetia kazinoki* which suppressed the proliferation of MCF‐7 breast and LNCaP prostate cancer cells, due to its capability to induce ROS-dependent cell death in gastric cells (Wei et al., 2011). Later on, it was shown that kazinol Q inhibits the growth of MCF‐7 breast and LNCaP prostate cancer cells, in part, through apoptosis induction (Weng et al., 2014). The antiproliferative activity of kazinol Q was found to be due at least in part to its inhibition of DNMT1 with an IC_50_ value of 7 µM (Weng et al., 2014). It is worth to mention that kazinol Q was the only active compound among the 12 isolated from formasan plants and used in this study.

### Resveratrol

Resveratrol (3, 4′, 5-trihydroxystilbene) (**5**) is a naturally occurring phytoalexin presents in grapes, berries, soy beans, pomegranate and peanuts, produced by spermatophytes, in response to an injury (Sinha et al., 2016). The source of resveratrol in the human diet is red wine (Cal et al., 2003). This compound has been shown to inhibit cancer initiation, promotion, and progression (Jang et al., 1997). Resveratrol has been reported to inhibit the activity of DNMT3b and DNMT1 in mammary tumors in a dose-dependent fashion (Qin et al., 2005; Qin et al., 2014). The combination of resveratrol with pterostilbene, another stilbene found in the plant, revealed a decrease of the activity of DNMT3b in HCC1806 triple-negative cancer cells without affecting the control MCF10A breast epithelial cell line (Kala et al., 2015). Another study showed that resveratrol can down-regulate the activity of DNMTs, as well as other proteins including HDAC1 and MeCP2 in MDA-MB-231 and MCF7 breast cancer cell lines (Mirza et al., 2013). However, the positive effects of resveratrol have been largely demonstrated *in vitro* or animals studies, with only limited effects reported in clinical studies (Shih et al., 2019). Furthermore, a lower DNMT inhibition of resveratrol has been observed when compared to other dietary bioactive compounds such as EGCG.

### Nanaomycin A

Nanaomycin A (**6**) is a quinone isolated from *Streptomyces rosa* var. *notoensis* and first reported as an antibiotic (Tanaka et al., 1975). The treatment of three different human tumor cell lines including HCT116 (colon), A549 (lung), and HL60 (bone marrow) cells with nanaomycin A resulted in growth inhibition of all three cell lines and caused induction of apoptosis (Kuck et al., 2010). Furthermore, an enzymatic assay revealed that the antiproliferative activity of nanaomycin A was due to its specific inhibition of DNMT3b with an IC_50_ value of 500 nM. This result was confirmed further by docking studies, which established the non-covalent binding of nanaomycin A to the active site of DNMT3b (see *Molecular Modeling and Docking as a Tool for a Mode of Action Prediction of Natural Product DNMTs and HDACs Inhibitors*) (Kuck et al., 2010). Furthermore, Nakamae and co-workers, recently suggested that, DNMT3b inhibition by nanaomycin A can promote the hepatoblast differentiation (Nakamae et al., 2018). The later result should assist in the future generation of functional hepatocyte-like cells for pharmaceutical research. Moreover, the induction of genomic demethylation is caused by nanaomycin A which is the first selective inhibitor of DNMT3b (Caulfield and Medina-Franco, 2011). It has been hypothesized that the inhibition arises from the nucleophilic attack of the catalytic cysteine residue within the active site of DNMT to the α,β-unsaturated carbonyl of nanaomycin A (Caulfield and Medina-Franco, 2011). While the further characterization is needed, the reactivity of the Michael acceptor moiety and toxicity of nanaomycin A may lead to challenges in such characterization in the future.

### Genistein

Genistein (**7**) is an isoflavone originally isolated from the dyer´s broom *Genista tinctoria* (Mukund et al., 2017). It was reported to be a potent inhibitor of cell proliferation on pancreatic cancer cell lines through apoptosis induction, regulation of the signal transducer and activator of transcription 3 (STAT3) signaling pathway, and G_0_/G_1_ cell cycle arrest (Bi et al., 2018). Ralhan and co-workers showed that genistein is able to induce a significant decrease in the transcript levels of all the DNMTs including DNMT1, DNMT3a, and DNMT3b, in breast cancer (Mirza et al., 2013). Another study confirmed this antiproliferative activity on the human breast cancers lines MCF-7 and MDA-MB-231 (Xie et al., 2014). The latter activity was found to be due to the inhibition of DNMT1 and molecular modeling indicated the direct interaction of genistein with the catalytic domain of this enzyme (Xie et al., 2014). However, only 40% inhibition of DNMT activity was observed at 100 mM contradicting the modeling data and thus indicating a low potential of genistein for DNMT inhibition. This weak effect suggests an indirect pathway of inhibition of DNA methylation, based on the identification of multiple targets for genistein including HDAC, tyrosine specific protein kinase, topoisomerase I and II, and NF-kB (Akiyama et al., 1987; Okura et al., 1988; Li and Sarkar, 2002; Sundaram et al., 2018). Despite the preclinical data reported for genistein showing its remarkable efficacy against prostate cancer *in vitro* with diverse molecular targets, there is no convincing clinical proof or evidence that genistein might be useful in prostate cancer therapy. Moreover, genistein is a multi-target compound limiting, therefore, its potential clinical use (Majid et al., 2009; Parker et al., 2009).

### Sulforaphane

The isothiocyanate sulforaphane (SFN) (**8**) is found in cruciferous vegetables, such as broccoli sprouts and broccoli (Fahey et al., 1997). The strong inhibition of the growth of the human breast cancer cells lines MCF-7, MDA-MB-231, and SK-BR-3 by sulforaphane with IC_50_ values of 14.05, 19.35, and 16.64 µM respectively has been reported. Inhibition of cell growth was accompanied by cell cycle arrest, elevation in the levels of the tumor suppressors p21 and p27 and cellular senescence as well as induction of apoptosis. The anticancer effects of sulforaphane were found to be mediated by a global DNA hypomethylation, decreased levels of DNMT1 and DNMT3b and changes in the microRNA profiles of the three breast cancer cells lines (Lewinska et al., 2017). A further study showed that combination of sulforaphane and withaferin A, another natural compound, significantly causes down-regulation of overexpressed DNMT3a, DNMT3b, and HDAC1 and breast cancer cell death (Royston et al., 2017). Recently, it has been demonstrated that sulforaphane can suppress the growth of NPC cells via the inhibition of DNMT1 and the restoration of the expression of Wnt inhibitory factor 1 (WIF1) (Chen et al., 2019b). Na and co-workers reported that sulforaphane up-regulates NrF2 expression and promotes its nuclear translocation through decreasing levels of DNA methylation of the Nrf2 promoter in a cellular model of Alzheimer's disease (Zhao et al., 2018). Furthermore, sulforaphane was shown to exert its chemopreventive effect in lung cancer A549 cells partly through the down-regulation of the activity of DNMT3a (Gao et al., 2018). Reduction of the toxicity of the chemotherapeutic drug cadmium selenide by sulforaphane in human hepatocytes through induction of glutathione synthesis was shown, thereby protecting the liver against toxicity and allowing the use of higher doses (Wang et al., 2015). However, there is no proof that a higher concentration could be achieved clinically using either diet-derived or supplemented SFN. The use of phytochemicals including SFN for patients with diagnosed cancers still need deep studies whether or not the patient is undergoing chemotherapy (Houghton, 2019). Furthermore, the presence of a highly electrophilic chemical functionality will probably cause several off-target effects despite that the downstream epigenetic effects observed upon the use of sulforaphane are promising and may improve chemopreventive activity (Cherblanc et al., 2013).

### Boswellic Acids

Boswellic acids (BA) (**9**) are pentacyclic terpenoids extracted from *Boswellia serrata*; a plant used traditionally to treat inflammatory diseases (Zhou et al., 2017). Inhibition of cell proliferation and apoptosis induction in colorectal cancer (CRC) cell line through up-regulation of miR-34a and down-regulation of miR-27a by the most active boswellic acid, acetyl-11-keto-β-boswellic acid (AKBA), is well established (Takahashi et al., 2012; Toden et al., 2015). AKBA induces demethylation and concurrent up-regulation of tumor suppressor genes including SAMD14 and SMPD3 in CRC cells (Shen et al., 2012). Moreover, AKBA was reported inhibiting DNMT in SW48 and SW480 CRC cell lines at a concentration of 40 µM. BA has been established as a multitargeting agent involved in the treatment of diverse chronic diseases including cancers (Roy et al., 2019). Indeed, modulation by boswellic acids of diverse molecular targets, such as kinases, enzymes, growth factors, receptors, transcription factors, and others related to the proliferation and survival of cells is possible (Roy et al., 2016). However, the possible development of boswellic acids as an effective drug has been tumbled down due to concerns regarding the pharmacokinetic properties.

### Z-Ligustilide

Z-ligustilide (**10**) is the most potent bioactive component found in *Angelica sinensis*, a herb from traditional Chinese medicine (TCM) used in the treatment of breast cancer (Ma et al., 2017). This compound has been reported to induce apoptotic cell death in human ovarian cancer cells (Lang et al., 2018). Z-ligustilide was reported to inhibit the growth of murine prostate cancer TRAMP C1 cells. The same study showed that Z-ligustilide reduced the methylation level of the first five CpGs of the NrF2 promoter. An enzymatic assay showed that Z-ligustilide blocks DNA methyltransferase activity of the CpG methylase M.SssI *in vitro* (Su et al., 2013). The latter is structurally significantly similar to the DNMT. This result suggests that Z-ligustilide acts through an indirect mechanism of DNA methylation inhibition limiting its potential use in clinical trials.

### Parthenolide

Parthenolide (**11**) is a germacrane sesquiterpene lactone isolated from the plant *Tanacetum parthenium* (Freund et al., 2019). This plant is commonly used for its inflammatory properties and suggested to be used in epigenetic cancer therapy (Akihisa et al., 2003; Ghantous et al., 2012). Parthenolide inhibits the detyrosination of microtubules and accelerates neuronal growth (Freund et al., 2019). A recent report showed that parthenolide induces apoptosis and inhibits proliferation of human 786-O kidney cancer cells *in vitro* (Dong et al., 2019). The compound is also known to induce apoptosis in primary acute myeloid leukemia (AML) cells, including the stem and progenitor cell compartment through inhibition of NF-kB and HSP70 (Pei et al., 2009). Parthenolide has been reported to inhibit DNMT1 with an IC_50_ value of 3.5 µM. This inhibition is thought to be due to its gamma methylene lactone which probably alkylates the proximal thiolate of Cys^1226^ of the catalytic domain (Liu et al., 2009a). Recently, a new parthenolide derivative, dimethylamino-parthenolide, has been reported to inhibit the Nuclear chain factor kappa‐light‐chain enhancer of activated B cells (NF-κB) pathway and causes depletion of glutathione levels; the latter causing cancer cells to be more susceptible to oxidative stress‐induced cell death (Pei et al., 2009; Lamture et al., 2018). This result highlights the potential role of this drug as a chemopreventive agent and in epigenetic cancer therapy. Recently, it has been demonstrated that combination of the anticancer drug actinomycin-D, which functions by intercalating into DNA, and dimethylamino-parthenolide results in a synergistic inhibition of Panc‐1 pancreatic cancer cell growth (Lamture et al., 2018). However, the potential clinical use of parthenolide is still not clear. Indeed, analysis of parthenolide activity at Cancer Research Technologies, using a fluorescence intensity assay for DNMT1 did not find DNMT1 activity (Cherblanc et al., 2013). Moreover, the presence of several off-target effects complicates the analysis of cell-based assays.

### Antroquinonol D

Antroquinonol D (**12**) is an ubiquinone derivative isolated from the mycelium of *Antrodia camphorata* (Wang et al., 2014). This compound inhibited the growth of MCF7, T47D, and MDA-MB-231 breast cancer cells without harming normal MCF10A and IMR-90 31 cells with IC_50_ values of 8.01, 3.57, and 25.08 μM respectively (Wang et al., 2014). The authors reported that antroquinonol D can inhibit the activity of DNMT1 in MDA-MB-231 breast cancer cells with an IC_50_ value lower than 5 µM. This result was confirmed by molecular modeling which revealed that antroquinonol D binds to the catalytic subunit of DNMT1 and competes for the same binding pocket in the DNMT1 enzyme as the cofactor SAM (S-adenosylmethionin) (Wang et al., 2014). Antroquinonol D was also found to reverse the silencing of multiple tumor suppressor genes in the same study.

## HDAC Inhibitors

Histone deacetylases (HDACs) also called lysine deacetylases (KDAC) are a family of hydrolases catalyzing removal of acetyl groups from lysine residues on histone tails (Glozak and Seto, 2007). This removal of acetyl groups allows compacted chromatin to reform and this process is associated with transcriptional repression (Glozak and Seto, 2007). HDACs are classified in four distinct subtypes: class I (HDACs 1, 2, 3, and 8 localized in the nucleus), class II (IIa: HDACs 4, 5, 7, and 9; IIb: HDACs 6 and 10, found in both nucleus and cytoplasm), class III (nicotinamide adenine dinucleotide-dependent SIRT [sirtuin] enzymes [Sirt 1–7]), and class IV (HDAC 11). The later shares structural similarities with both class I and II HDACs as they are Zn-dependent enzymes (Haberland et al., 2009; McKinsey, 2012). Class I HDACs play an important role in cell survival and proliferation, while class II may have tissue-specific roles (Li et al., 2015). It has been reported that functional dysregulation of HDACs affects the expression of numerous genes that have an impact on apoptosis and the cell cycle (Hauser and Jung, 2008). Indeed, HDAC1 is overexpressed in prostate cancer cells, while gastric carcinomas, colorectal carcinomas, and cervical dysplasias overexpress HDAC2 (Halkidou et al., 2004; Huang et al., 2005; Song et al., 2005).

HDAC inhibitors (HDACi) are emerging therapeutic agents, since their targets play an important role in cancer initiation and progression (Lee et al., 2017). Indeed, histone deacetylation plays a key role in tumor suppressor genes silencing (TGSs) in several cancers, thus the restoration of the acetylation of lysine residues by HDAC inhibitors will oppose the frequent HDAC overexpression in cancer (Perri et al., 2017). HDACs are also known to regulate non-histone proteins involved in cancer development such as p53, and NF-kB and can affect their function leading to modified expression of cancer-related genes (Saunders and Verdin, 2007; Wilcox, 2016). HDACs can repress the expression of receptors for growth-restraining signaling molecules such as TGFβ receptor (transforming growth factor beta receptor), leading to unhindered cell growth, which is preventable by HDAC inhibition (Glozak and Seto, 2007).

**Figure 2:**
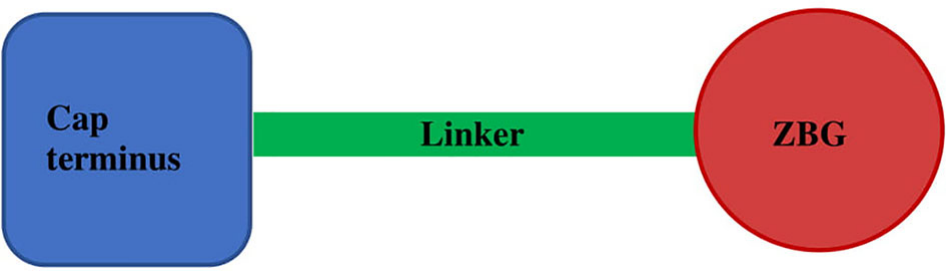
Common features of HDAC inhibitors.

The well-known effect of HDACi is to lead to cell cycle arrest and induction of cell senescence (Gołąbek et al., 2015). Since the approval of suberanilohydroxamic acid (SAHA) by the US Food and Drug Administration for the treatment of T-cell lymphoma in 2006, the relevance of HDACi in cancer therapy has been strongly pointed out. HDACi are usually classified in two main groups: group I which displays the zinc-binding mode of action includes linear inhibitors, cyclic tetrapeptides, and cyclic depsipeptides; and group II with a non-zinc-binding mode of action includes miscellaneous inhibitors (Lascano et al., 2018). It is worth mentioning that most HDACi share the same overall structure including a cap terminus, a linker region, and a zinc-binding group (ZBG) (**Figure 2**). As shown in **Table 2**, nature is a promising source of HDACi that may inspire the development of lead structures for the potential treatment of cancer. However, the lack of HDAC isoform selectivity for most of the natural products may limit their clinical use.

**Table 2:** Selected HDAC inhibitors.

Compound name	IC_50_ Values	HDAC Isoforms	Reference
Trichostatin A	0.08 µM	NT	Okamoto et al., 2006
Depudecin	4.70 µM	HDAC1	Kwon et al., 1998
Psammaplin A	0.042 µM	HDAC1	Baud et al., 2012
Sulforaphane	36.00 µM	HDAC2	Choi et al., 2018
	0.60 µM	HDAC9	
Bis(4-hydroxybenzyl)sulfide	1.43 µM	NT	Son et al., 2007
Azumamide E	0.05 µM	HDAC1	Maulucci et al., 2007
	0.10 µM	HDAC2	
	0.08 µM	HDAC3	
Apicidin	0.001 µM	NT	Singh et al., 2002
Apicidin A	0.001 µM	NT	
Apicidin D_1_	0.004 µM	NT	
Apicidin B	0.01 µM	NT	Singh et al., 2001
Apicidin C	0.006 µM	NT	
FR235222	0.017 µM	NT	Sasamura et al., 2010
AS1387392	0.022 µM	NT	
Chlamydocin	0.00015 µM	HDAC1	Furumai et al., 2001
1-alaninechlamydocin	0.0064 µM	NT	Du et al., 2014
Trapoxin A	0.00082 µM	HDAC1	Furumai et al., 2001
	0.524 µM	HDAC6	
Microsporin A	0.55 µM	HDAC8	Gu et al., 2007
Romidepsin	0.036 µM	HDAC1	Furumai et al., 2002
	0.047 µM	HDAC2	
	0.510 µM	HDAC4	
Largazole	0.0114 µM	HDAC1	Souto et al., 2010
	3.0 µM	HDAC4	
Epicocconigrone A	4.6 µM	HDAC6	El Amrani et al., 2014
	1.6 µM	HDAC8	
	8.4 µM	HDAC10	

NT, Not Tested.

### Zinc-Binding Inhibitors

#### Linear HDAC Inhibitors (**Figure 3**)

##### Trichostatin A

Trichostatin A (TSA) (**13**) is the first natural product derived HDAC inhibitor and was isolated from the bacterium *Streptomyces hygroscopicus* (Tsuji et al., 1976; Yoshida et al., 1990). Its structure consists of an aromatic group, a conjugated diene linker region, and a hydroxamic tail. The first racemic synthesis of TSA was reported by Krebs and co-workers in 1983 and this compound was first reported to display antifungal activity (Tsuji et al., 1976; Fleming et al., 1983). Trichostatin A was reported to reversibly inhibit mammalian HDAC and accordingly was found to induce accumulation of acetylated histones in a variety of mammalian cell lines when applied in nanomolar concentration (Yoshida et al., 1990; Yoshida et al., 1995). The crystal structure showed that trichostatin A non-covalently binds to the active site of HDACs through the terminal hydroxamic acid group which chelates the Zn^2+^ in a bidendate fashion (see *Natural Product HDAC Inhibitor in the PDB*) (Finnin et al., 1999). Kinetic studies revealed that trichostatin A is a competitive inhibitor (Sekhavat et al., 2007). Yokoyama and co-workers reported that trichostatin A inhibits HDAC in a dose-dependent manner in vascular smooth muscle cells (VSMCs) with an IC_50_ value of 0.08 µM (Okamoto et al., 2006). Trichostatin A is frequently used as a positive control and a reference when compared to other HDAC inhibitors and considered today as one of the most potent HDAC inhibitors (Ying et al., 2008; Benelkebir et al., 2011). However, the observed mutagenicity of the hydroxamic moiety along with lack of HDAC isoform selectivity limits its clinical use (Khan et al., 2008; Shen and Kozikowski, 2016).

**Figure 3:**
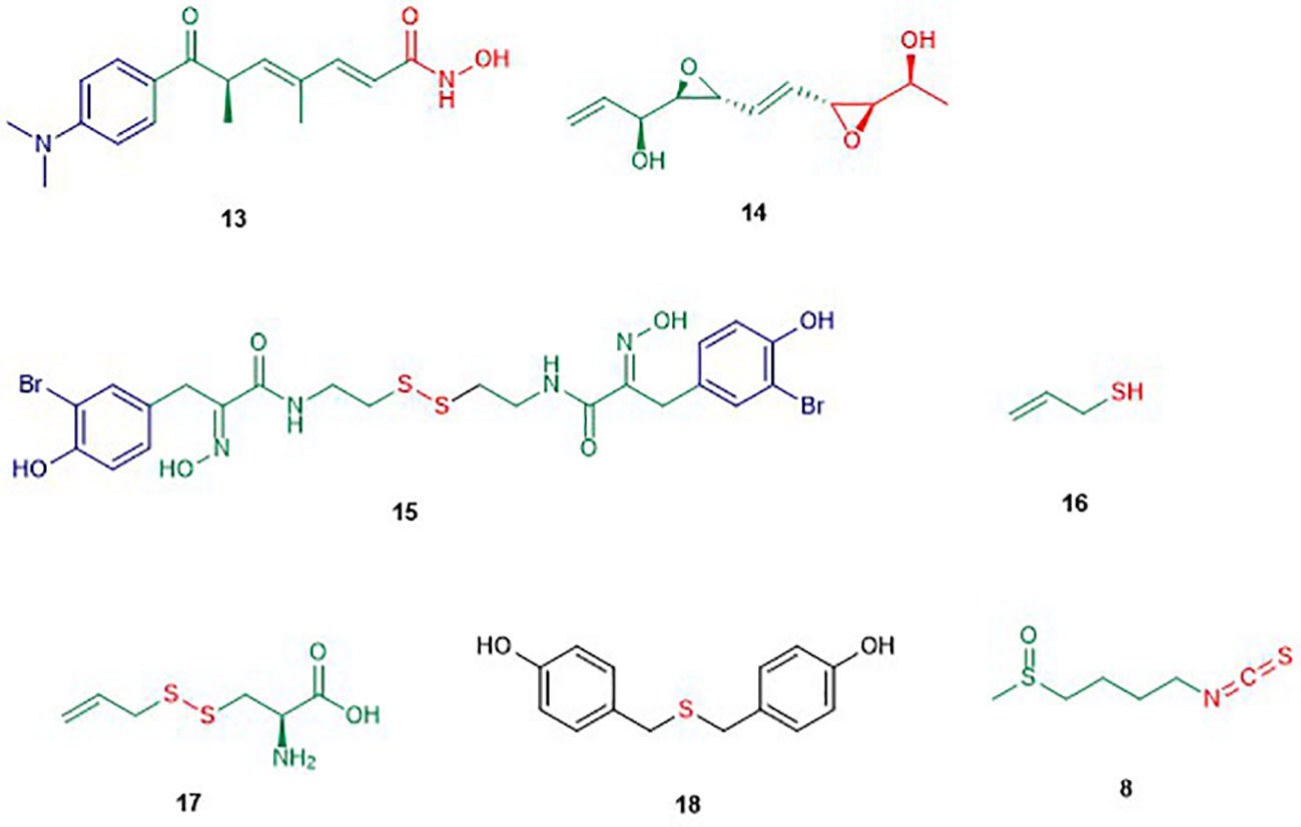
Structures of linear HDAC inhibitors. The colors are representative of different parts shown in **Figure 2**.

##### Depudecin

Depudecin (**14**) is a linear polyketide containing two epoxide groups isolated from the culture broth of the soil fungus *Alternaria brassiccicola* (Matsumoto et al., 1992). The structure of depudecin consists of a bis-*trans*-epoxide moiety and six asymmetric centers. Depudecin has been reported to display an *in vitro* inhibitory effect against HDAC1 in a dose-dependent manner with an IC_50_ value of 4.7 µM (Kwon et al., 1998). The relatively low potency of depudecin precluded further work on this compound. Depudecin showed also the ability to induce morphological reversion in NIH 3T3 fibroblast cells to their normal flat phenotype (Kwon et al., 1998).

##### Psammaplin A

Psammaplin A (PsA) (**15**) is a monobrominated tyrosine derived oxime containing cystamine isolated from several marine sponges including *Psammaplysilla* sp. (Quiñoà and Crews, 1987; Tabudravu et al., 2002). Psammaplin A is one of the members of the psammaplin class and was reported to suppress carcinogenic properties of several human cancer cell lines including lung, breast, colon, and ovarian cancer *in vitro* (Park et al., 2003). Psammaplin A inhibited *in vivo* tumor growth in the A549 lung xenograph mouse model while maintaining low toxicity (Piña et al., 2003). Moreover, psammaplin A was reported to inhibit the activity of HDAC and DNMT at nanomolar levels *in vitro* (Piña et al., 2003). Hyperacetylation of histone H3 by psammaplin A demonstrated that the compound is a specific and potent inhibitor of class I HDAC rather than class II (Kim et al., 2007). Moreover, psammaplin A was found to be a natural prodrug. Its activation is caused by reduction of the disulfide bond leading to a thiol. The latter thiol chelates the Zn^2+^ ion in the active site of HDAC and precludes access to the natural substrate (Kim et al., 2007). Indeed, the reduced form of psammaplin A highly selectively inhibited HDAC1 with an IC_50_ value of 45 nM (Baud et al., 2012). The hyperacetylation of histone by psammaplin A was correlated with an up-regulation of tumor suppressors such as p21^WAFI^ and gelsolin (Kim et al., 2007; Ahn et al., 2008). Psammaplin A was also reported to induce an increase of apoptosis, most likely by inducing expression of p21^WAF1^. It is worth to mention that the physiologic instability of the psammaplin class has precluded further clinical investigations. However, an analogue of psammaplin class, NVP-LAQ824, which induces apoptosis, has entered phase I clinical trials (Remiszewski, 2003; Cuneo et al., 2007).

##### Diallyl Disulfide (DADS)

Diallyl disulfide compounds are organosulfur compounds (OSC) which are released by plants of the *Allium* genus including onion, garlic, scallion, and leek (Bae et al., 2019). In general, organosulfur compounds are known to modulate the activity of several enzymes involved in the activation or detoxification of carcinogens and inhibit the formation of DNA adducts in diverse target tissues (Omar and Al-Wabel, 2010). DADS are known to induce cell cycle arrest, differentiation, and apoptosis in several cancer cell lines (Herman-Antosiewicz and Singh, 2004). In fact, DADS belong to the group of dietary HDAC inhibitors. Allyl mercaptan (**16**), a well-known DADS, induced histone acetylation in the liver and this compound was also recognized as the active HDAC inhibitor rather than the parent compound DADS (Druesne et al., 2004). Indeed, the authors reported that allyl mercaptan inhibits 92% of HDAC activity while the parent compound, inhibited only 29% at the concentration of 200 µM. DADS is also known to induce the hyperacetylation of histone H4, the activation of caspase-3, and the modulation of the anti-apoptotic paralogues including Bcl2, BAX ,and Bcl-xL in human leukemia, lung cancer, and breast cancer (Herman-Antosiewicz and Singh, 2004; Zhao et al., 2006). The latter result suggested that the Bcl-2 family is targeted by DADS. Besides, DADS was reported to cause hyperacetylation of H3 and H4 leading to an increase of the expression of the tumor suppressor p21^WAFI^ in human acute myeloid leukemia HL-60 cells and colon cancer cells *in vitro* and *in vivo*.

*S*-allyl-mercapto-*L*-cysteine (SAMC) (**17**) is another organosulfur compound which was also reported to induce hyperacetylation of H3 and H4 in human colon and breast cancer cells (Lea et al., 2002). The same study reported that SAMC inhibits cell proliferation of DS19 mouse erythroleukemia cells with an IC_50_ value of 0.5 µM (Lea et al., 2002). SAMC has also been reported to inhibit the growth of the breast cancer cell lines MCF-7 and MDA-MB-231 through cell cycle arrest in the G_0_/G_1_ phase (Zhang et al., 2014). These findings support the continue investigation of SAMC as an alternative agent in the chemoprevention and chemotherapy of human breast cancer. Despite the lack of direct inhibition of HDAC by SAMC, it can be assumed that this compound displays this activity with the same mechanism as other organosulfur compounds. However, the stability of DADS causes difficulties in reproducibility of findings.

##### Sulforaphane (SFN)

The isothiocyanate sulforaphane (SFN) (**8**), as mentioned earlier, is found in cruciferous vegetables, including broccoli sprouts and broccoli (Fahey et al., 1997). Isothiocyanates result from the hydrolysis of glucoraphanin by the plant enzymes myrosinases (Kim and Park, 2016). Isothiocyanates are a family of compounds including sulforaphane, allyl isothiocyanate, benzyl isothiocyanate, phenetyl isothiocyanate, etc. (Kim and Park, 2016). Sulforaphanes have been reported to possess anticancer activities in xenograft models of prostate cancer and in induced animal models (Zhang et al., 1994; Fahey et al., 1997). Myzak and co-workers reported that sulforaphane possesses anticancer activity through the inhibition of HDAC activity and increase in the histone acetylation in HCT116 human colorectal cancer cells (Myzak et al., 2004). The same study showed that the metabolite sulforaphane-cysteine which displayed greater HDAC inhibitory effect than SFN at a concentration of 15 µM is the active form of SFN and other isothiocyanates. This observation was further confirmed by molecular studies which revealed a plausible interaction for sulforaphane-cysteine within the active site of the HDAC-like protein (see *Natural Product HDAC Inhibitor in the PDB*) (Myzak et al., 2004). Moreover, 40% of the growth inhibition of xenografts of human PC-3 prostate cancer cell in mice by SFN was observed at a concentration of 15 µM, accompanied by as significant down-regulation of HDAC activity in the xenografts (Myzak et al., 2007). Recently, SFN has been reported to selectively inhibit HDAC2 and 9 with IC_50_ values of 36 and 0.6 µM respectively (Choi et al., 2018). *In silico* studies confirmed that isothiocyanates can bind to allosteric active sites of HDAC based on their similar structural features with other HDAC inhibitors (Furumai et al., 2001; Ho et al., 2009; Rajendran et al., 2013). However, isothiocyanates are known to react preferably with diverse thiol and amine (bio)nucleophiles (Jacob, 2006), which strongly suggest non-specific and likely indirect effects on a given epigenetic target.

##### Bis(4-Hydroxybenzyl)sulfide

Bis(4-hydroxybenzyl)sulfide (**18**) is a sulfur compound isolated from the root extract of the Chinese medicinal plant *Pleuropterus ciliinervis* (Son et al., 2007). *In vitro*, it displayed inhibitory activity against HDAC in HeLa cells with an IC_50_ value of 1.43 µM (Son et al., 2007). Besides, this compound inhibited also the growth of several cancer cells lines among which the prostate PC-3 and breast MDA-MB-231 cell line with IC_50_ values of 7.86 and 1.45 µM respectively (Son et al., 2007). Like other organosulfur compounds, stability is a big issue that may affect the reproducibility of these findings.

#### Cyclic Tetrapeptides (**Figure 4**)

##### Azumamide E

The cyclic tetrapeptide azumamide E (**19**) was isolated from the marine sponge *Mycale izuensis* along with other azumamides (A, B, C, and D) (Nakao et al., 2006). Structurally, azumamide E include four *D*-α-amino acids (*D*-Phe, *D*-Tyr, *D*-Ala, *D*-Val) and a unique *β*-amino acid assigned as the 13-membered macrocycle [(*Z*)-(2*S*,3*R*)-3-amino-2-methyl-5-nonenedioc acid] (known as Amnaa). Fusetani and co-workers reported that azumamide E possesses a strong inhibition of HDACs in K562 human leukemia cells, with an IC_50_ value of 0.064 µM (Nakao et al., 2006). Another study reported that azumamide E selectively inhibited class I HDAC in HeLa nuclear extracts, particularly HDACs 1-3 with IC_50_ values of 0.05, 0.1, and 0.08 µM respectively (Maulucci et al., 2007). Docking studies showed that azumamide E inserts the Amnaa side chain into the active site of HDACs, where the carboxylic acid will chelate the Zn^2+^ in a bidentate fashion (Maulucci et al., 2007). Total synthesis of azumamide E revealed that switching of the carboxylic acid group to a hydroxamic acid leads to an increase in potency for HDAC inhibition (Wen et al., 2007). A subsequent synthesis of azumamide E by Villadsen et al., revealed that **19** is also a potent inhibitor of HDAC10 and 11 evidencing its lack of isoform specificity (Villadsen et al., 2013).

**Figure 4:**
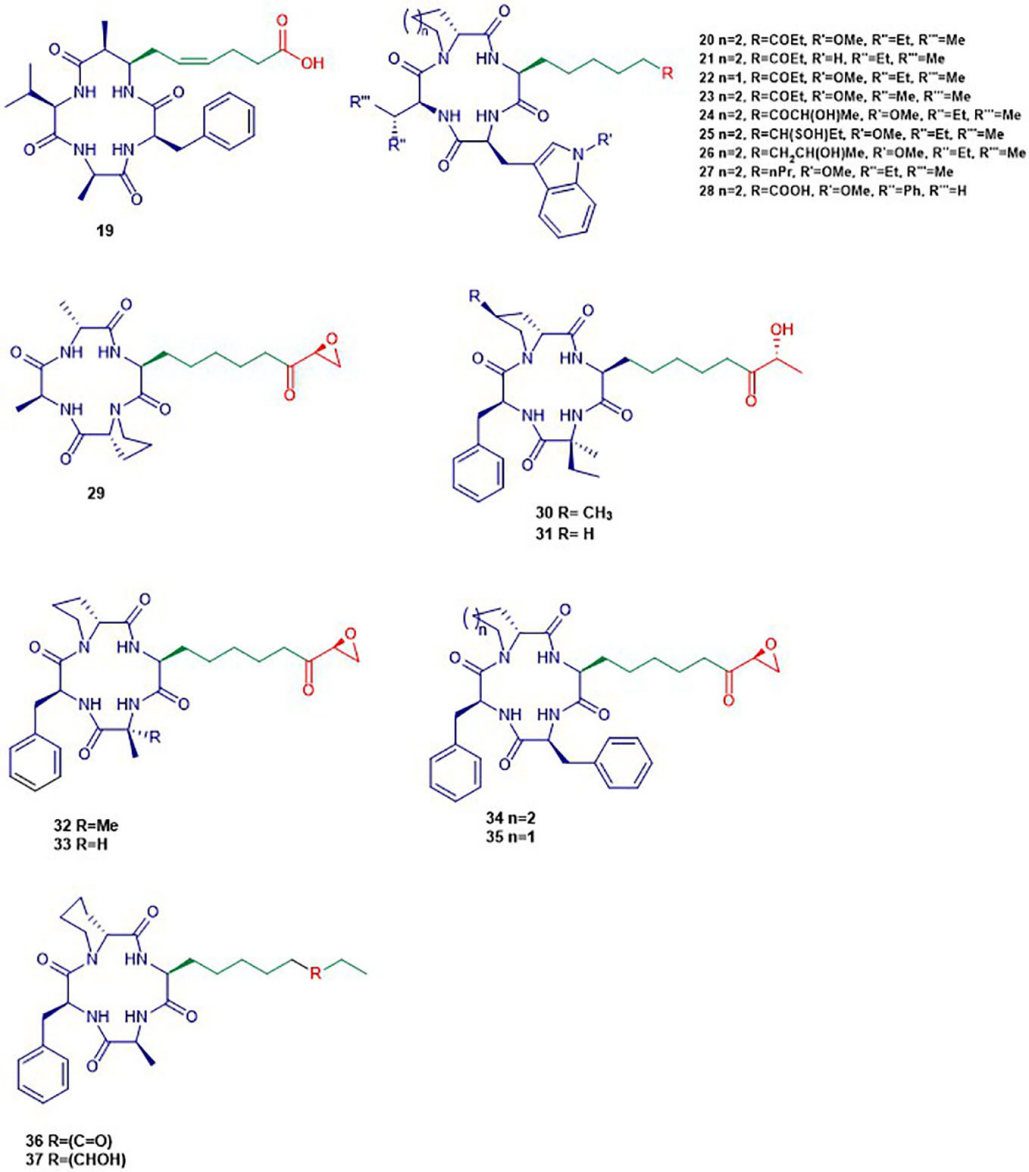
Structures of cyclic tetrapeptides. The colors are representative of different parts shown in **Figure 2**.

##### Apicidins

Apidicin (**20**) and apidicin A (**21**) are a family of natural products first isolated from the endophytic fungus *Fusarium pallidoroseum* (Singh et al., 1996). Structurally, all apicidins contain *N*-methoxy-*L*-tryptophan, except for apidicin A. Moreover, all apidicins contain *D*-pipecolinic acid, except for apidicin B (**22**) which contains *D*-proline. Furthermore, apidicin C (**23**) and F (**28**) contain *L*-valine and *L*-phenylalanine, respectively, while the other apidicins including apicidin D_1_ (**24**), D_2_ (**25**), D_3_ (**26**), and E (**27**) consist of *L*-isoleucine at the corresponding position. Another common feature of all the apidicins is the presence of (2*S*)-amino-8-oxo-decanoic acid (Aoda) or its derivative. These compounds displayed a high inhibitory activity of protozoal HDAC and in HeLa cell extracts in nanomolar range (Singh et al., 2001; Singh et al., 2002). A selective inhibition of class I HDAC2, 3, and 8 in nanomolar concentrations by apidicin A and D_1_ (**24**) has been reported as well as the inhibition of class IIa HDAC 4 and 7 in ovarian cancer cells (Khan et al., 2008; Ahn et al., 2012). The binding of apicidin to the active site of HDAC is enabled through the insertion of the acetylated lysine mimic. Indeed, the carbonyl group can chelate the Zn^2+^ at the bottom of the active site precluding the binding of the natural substrate as demonstrated by the lack of activity of apicidin D_2_ (**25**) and D_3_ (**26**) (Colletti et al., 2001; Singh et al., 2001). Apidicin inhibited the proliferation of diverse cancer cell lines through the induction of transcriptional activation of p21^WAFI/Cip1^ and gelsolin (Han et al., 2000; Kim et al., 2001). The reversion of morphological changes of HeLa cells as well as hyperacetylation of histone H4 accompanied antiproliferative activity of apidicin on those cells (Han et al., 2000). Apicidin-mediated growth inhibition of the promyelocytic leukemia cell line HL60 was also reported to be due to a transiently increased expression of Fas/Fas ligand. The latter resulted in activation of caspase-3 and 9 and execution of apoptotic cell death (Kwon et al., 2001).

##### Helminthosporium carbonum *(HC) Toxin*

HC-Toxin (**29**) is a cyclic tetrapeptide isolated from the fungal culture of *Helminthosporium carbonum* (Liesch et al., 1982). The structure of HC-toxin consists of *D*-proline, *D*-alanine, *L*-alanine, and (2*S*)-amino-8-oxo-9,10-epoxydecanoic acid (Aoe). HC-Toxin is an inhibitor of HDAC in several organisms including plants, insects, and mammals (Walton et al., 2006). Its Aoe moiety has been reported to be relevant for HDAC inhibition as well as the α-keto epoxide moiety (Walton and Earle, 1983). Indeed, the latter binds covalently into the active site of HDAC through epoxide opening by nucleophilic residues of the active site (Walton and Earle, 1983). HC-toxin was reported to induce G0/G1-cell arrest and apoptosis in neuroblastoma (NB) cell lines and primary cell cultures in the nanomolar range (Deubzer et al., 2008). In other studies, HC-toxin displayed antiproliferative activity against intrahepatic cholangiocarcinoma (ICC) cells and breast cancers cells lines (Joung et al., 2004; Zhou et al., 2016).

##### FR235222

FR235222 (**30**) was initially reported from the fermentation broth of the soil fungus *Acremonium* sp. No. 27082 (Mori et al., 2003). The structure of FR235222 consists of *L*-Phe and three unusual α-amino acids including (2*R*,4*S*)-4-*methylproline* (4-MePro), and (2*S*)-isovaline (iva), and (2*S*,9*R*)-2-amino-8-oxo-9-hydroxydecanoic acid (Aoh). This natural compound showed potent inhibition of partially purified HDAC fractions mammalian lymphoid cell lines (IC_50_ value of 17 nM) and selective immunosuppressive activity. Indeed, the authors found that FR235222 selectively inhibits both lymphocyte proliferation and lymphokine production; the target was identified as HDAC in T cells. The authors reported that FR235222 caused G1 cycle arrest accompanied by an increase of p21 and down-regulation of cyclin E, antiproliferative effects, and accumulation of acetylated histone H4. Another study, in which the prostate cancer cell line LNCaP was treated with FR235222 at a concentration of 0.5 µM, revealed that the increase in histone H4 acetylation is accompanied by caspase-3-dependent induction of apoptosis (D’Acunto et al., 2010). Besides, the authors demonstrated that FR235222 can increase the level of the endogenous anti-inflammatory protein ANXA1 involved in apoptosis. It is worth mentioning that, the restoration of ANXA1 expression in the prostate cancer cell line LNCaP reduced cell viability and proliferative response and induced caspase-mediated apoptosis (Hsiang et al., 2006). FR235222, with an IC_50_ value of 60 nM, was also reported to inhibit HeLa cell HDACs and HDAC3 was identified as its main target in *Toxoplasma* tachyzoites (Yoshida et al., 1990; Bougdour et al., 2009).

AS1387392 (**31**), a synthetic analogue of FR235222, in which the MePro of FR235222 is replaced by proline, was also isolated from the same fungus *Acremonium* sp. This compound showed similar HDAC inhibitory effects as FR235222 with IC_50_ an value of 22 nM (Sasamura et al., 2010). AS1387392 also displayed potent inhibitory activity against splenocyte proliferation, with an IC_50_ value of 4.6 nM (Sasamura et al., 2010).

##### Chlamydocin

Chlamydocin (**32**) was first isolated from the fungus *Diheterospora chlamydospria* and is a cyclic tetrapeptide containing 2-aminoisobutyric acid (Aib), *L*-phenylalanine, *D*-proline, and *L*-2-amino-8-oxo-9,10-epoxydecanoic acid (Closse and Huguenin, 1974). Chlamydocin exhibited cytotoxicity against mouse P-815 mastocytoma cells *in vitro* with a 10–100 times higher activity than clinical agents including actinomycin D, vinblastine, vincristine, amethopterin, and colchicine in the same assay (Stähelin and Trippmacher, 1974; Walton et al., 1985). Chlamydocin has also been reported to inhibit the proliferation of diverse cancer cell lines with IC_50_ ranging from 0.36 to 45 nM. In the same study, it was reported that chlamydocin inhibits HDAC with an IC_50_ value of 1.3 nM and the antiproliferative activity was found to be accompanied by an increase in the accumulation of the acetylated histones H3 and H4, the induction of p21^Wafi/Cip1^, and cell cycle arrest in the G_2_/M phase (Schepper et al., 2003). Chlamydocin selectively inhibited HDAC1 with an IC_50_ value of 0.15 nM (Furumai et al., 2001). Chlamydocin may share a similar mode of action as the aforementioned epoxide-based HDAC inhibitors such as HC-Toxin.

The fungal culture *Tolypocladium* sp. produced a closely related compound 1-alaninechlamydocin (**33**), which displayed strong antiproliferative effects in a human pancreatic carcinoma cell line MIA PaCa-2 in a nanomolar range (Du et al., 2014). Besides, 1-alaninechlamydocin also inhibited the growth of another human pancreatic carcinoma cell line (Panc-1) and the immortalized pancreatic duct cell line hTERT-HPNE at low-nanomolar concentration without induction of cytotoxicity in both cell lines at a concentration up to 10 µM. It was found that 1-alaninechlamydocin induces apoptosis and G2/M cell cycle arrest by inhibiting HDAC activity with an IC_50_ value of 6.4 nM (Du et al., 2014).

##### Trapoxin (TPX)

Trapoxin A (**34**) and B (**35**) are cyclotetrapeptides originally isolated from the fungal culture of *Helicoma ambiens* and caused the increase of highly acetylated core histones in diverse mammalian cell lines (Itazaki et al., 1990; Kijima et al., 1993). Trapoxin A (**34**) (also known as trapoxin) induced cell differentiation, cell cycle arrest, and reversal of transformed cells morphology (Yoshida et al., 1995). Trapoxin (**34**) was first reported to be an irreversible HDAC inhibitors (Taunton et al., 1996). However, Horinouchi and co-workers demonstrated later that trapoxin reversibly inhibits only HDAC6 through the ketone moiety. The latter undergoes a nucleophilic attack to form a zinc-bound tetrahedral gem-diolate without affecting the epoxide moiety (Furumai et al., 2001). This mechanism is in contrast to other epoxyketone that are commonly thought to be irreversible HDAC inhibitors (Taunton et al., 1996). In the same study, **34** displayed selective inhibition of HDAC1 and 6 with IC_50_ values of 0.82 and 524.0 nM respectively while **35** showed similar potency (Furumai et al., 2001). Also, trapoxin B (**35**) was reported to be more potent than trichostatin A in the respective inhibition of H1299 and HCT116 cell proliferation (Remiszewski et al., 2002). It has been reported that, trapoxin A (**34**) binds to the active site of HDAC8 through the same mode of action already known by its effect on HDAC6 (Porter and Christianson, 2017).

##### Microsporin A

Microsporin A (**36**) which is closely related to the trapoxins was produced by the fungus *Microsporum* cf. *gypseum* along with an analogue Microsporin B (**37**) (Gu et al., 2007). Both natural products harbor a nonproteinogenic moiety made of (2*S*)-amino-8-oxodecanoic acid (Aoda) and (2*S*)-amino-8-hydroxydecanoic acid respectively along with other units including *D*-pipecolinic, *L*-phenylalanine, and *L*-alanine (Gu et al., 2007). Microsporin A (**36**) showed potent *in vitro* cytotoxicity against human colon adenocarcinoma HCT-116 and a mean IC_50_ value of 2.7 µM in the National Cancer Institute’s diverse 60-cell line panel. Microsporin A (**36**) in the same study showed greater *in vitro* inhibition against both a mixture of HDACs and HDAC8 than the reference antitumor agent HDAC inhibitor SAHA with IC_50_ values of 0.14 and 0.55 µM, respectively (Gu et al., 2007).

#### Cyclic Depsipeptides (**Figure 5**)

##### Romidepsin

The bicyclic depsipeptide romidepsin (**38**) was originally produced by *Chromobacterium violaceum* that displayed antitumor activity (Ueda et al., 1994). This natural product was initially isolated under the name FR901228 and is now adays known as romidepsin or Istodax^R^ (trade name), or FK228 (Vandermolen et al., 2011). In 2009, the prodrug romidepsin (**38**) was approved for the treatment of cutaneous and/or peripheral T-cell lymphoma by the US Food and Drug Administration (FDA) (Prince et al., 2013). The chemical structure of **38** consists of two valine units with opposite configurations, *D*-cysteine, (*Z*)-dehydrobutyrine, and (3*S*)-hydroxy-7-mercapo-4-heptenoic acid (Shigematsu et al., 1994). Compound **38** acts as a potent and selective HDAC inhibitor of class I HDACs in particular HDAC1, 2, and 4 with IC_50_ values of 36, 47, and 510 nM respectively (Furumai et al., 2002). This compound has an internal disulfide bond of **38** that is reduced in the presence of cellular glutathione to generate a sulfhydryl (thiol) moiety, which is the active form of Romidepsin behaving as a Zn^2+^ binding group in the active site.

**Figure 5:**
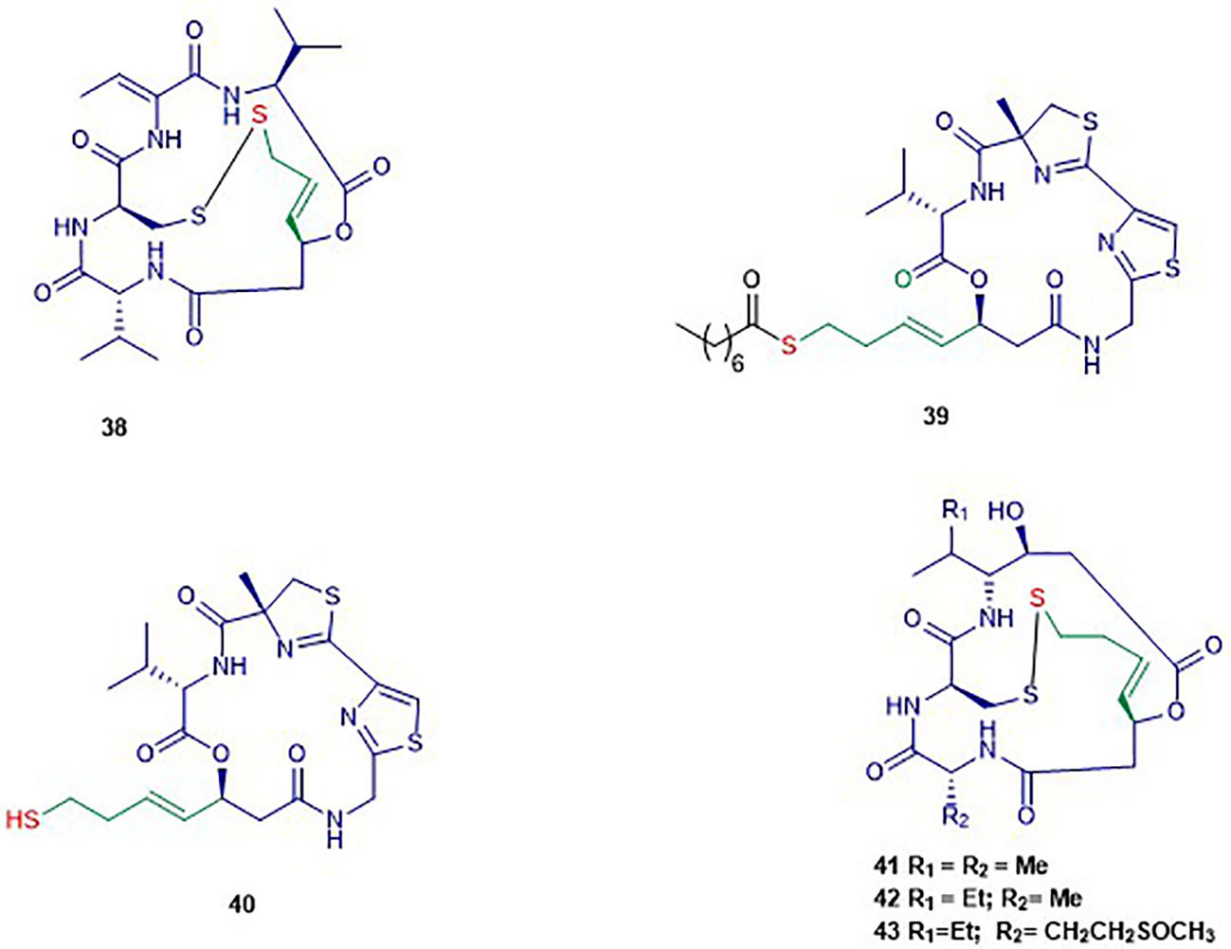
Structures of cyclic depsipeptides. The colors are representative of different parts shown in **Figure 2**. The black color is representative of the product moiety.

##### Largazole

Largazole (**39**) was isolated through a bioassay-guided fractionation of the crude extract of the cyanobacterium *Symploca* sp. (Taori et al., 2008). Its structure consists of uncommon features including a thiazole ring linearly fused to a substituted (4*R*)-methylthiazoline and a (3*S*)-hydroxy-7-mercaptohept-4-enoic acid like in FK228. Largazole (**39**) selectively inhibited, at nanomolar concentration, the growth of the human epithelial cancer cells MDA-MB-231 in a dose-dependent manner. Indeed, non-transformed murine epithelial cells NmuMG remained unsusceptible to **39** in this study (Taori et al., 2008). The authors reported the same selectivity for transformed fibroblastic osteosarcoma U2OS cells (IC_50_ 55 nM) over nontransformed fibroblast NIH3T3 (IC_50_ 480 nM). It has been reported that **39** selectively inhibits class I HDAC1 over class II HDAC4 with IC_50_ values of 11.4 nM and 3 µM respectively (Souto et al., 2010). Largazole induced an increase of H3 and α-tubulin acetylation, and an up-regulation of p21^WAF1CIP1^ in NB4 cells (Souto et al., 2010). Increased acetylation of H3 and up-regulation of p21^WAF1XIP1^ was also observed in another study, in which **39** induced cell cycle arrest at G_1_ phase at nanomolar range concentration in HCT116 cells (Liu et al., 2010). Compound **39** is a prodrug that requires activation through hydrolysis to form the thiol **40** as the active form exhibiting high isozyme-selective HDAC inhibition activity (Hong and Luesch, 2012).

##### Spiruchostatins

Spiruchostatin A (**41**) and B (**42**), both sharing several structural features with romidepsin, were isolated from *Pseudomonas* sp. in 2001 (Masuoka et al., 2001). Another analogue, spiruschostatin C (**43**), was isolated from *Burkholderia thailandensis* (Masuoka et al., 2001; Klausmeyer et al., 2011). Spiruchostatin A–C (**41–43**) demonstrated selective inhibition of class I HDACs, in particular HDAC1, with low nanomolar IC_50_ values (Narita et al., 2009; Narita et al., 2013). Moreover, **41** was reported inhibiting 14 cancer cell lines at nanomolar range (Shindoh et al., 2008). In the latter study, it was found that **41** induces selective accumulation of acetylated histones in tumor tissues, p21^WAFI/Cip1^ expression and cell cycle arrest. Another study reported that a higher increase in the formation of intracellular reactive oxygen species accompanied induction of apoptosis in human lymphoma U937 cells by **41** and **42** (Rehman et al., 2014).

### Non Zinc-Binding Inhibitors (**Figure 6**)

#### Ursolic Acid

The pentacyclic ursolic acid (**44**) can be found in fruits such as blueberries, apple peels, olive, and cranberries as well as in diverse herbs (Ikeda et al., 2008). Compound **44** was reported to inhibit the growth of HL60 cells resulting from an increase in the accumulation of acetylated histone H3 (Chen et al., 2009). The increased acetylation of H3 was found to be induced by the inhibition of HDAC 1, 3, 4, 5, and 6 (Chen et al., 2009). The binding of **44** to the active site of class I HDAC and HDAC7 isoforms has been recently confirmed through docking studies (Ishola and Adewole, 2019). Moreover, the same study showed **44** fulfills oral druggability of Lipinski rule five.

**Figure 6:**
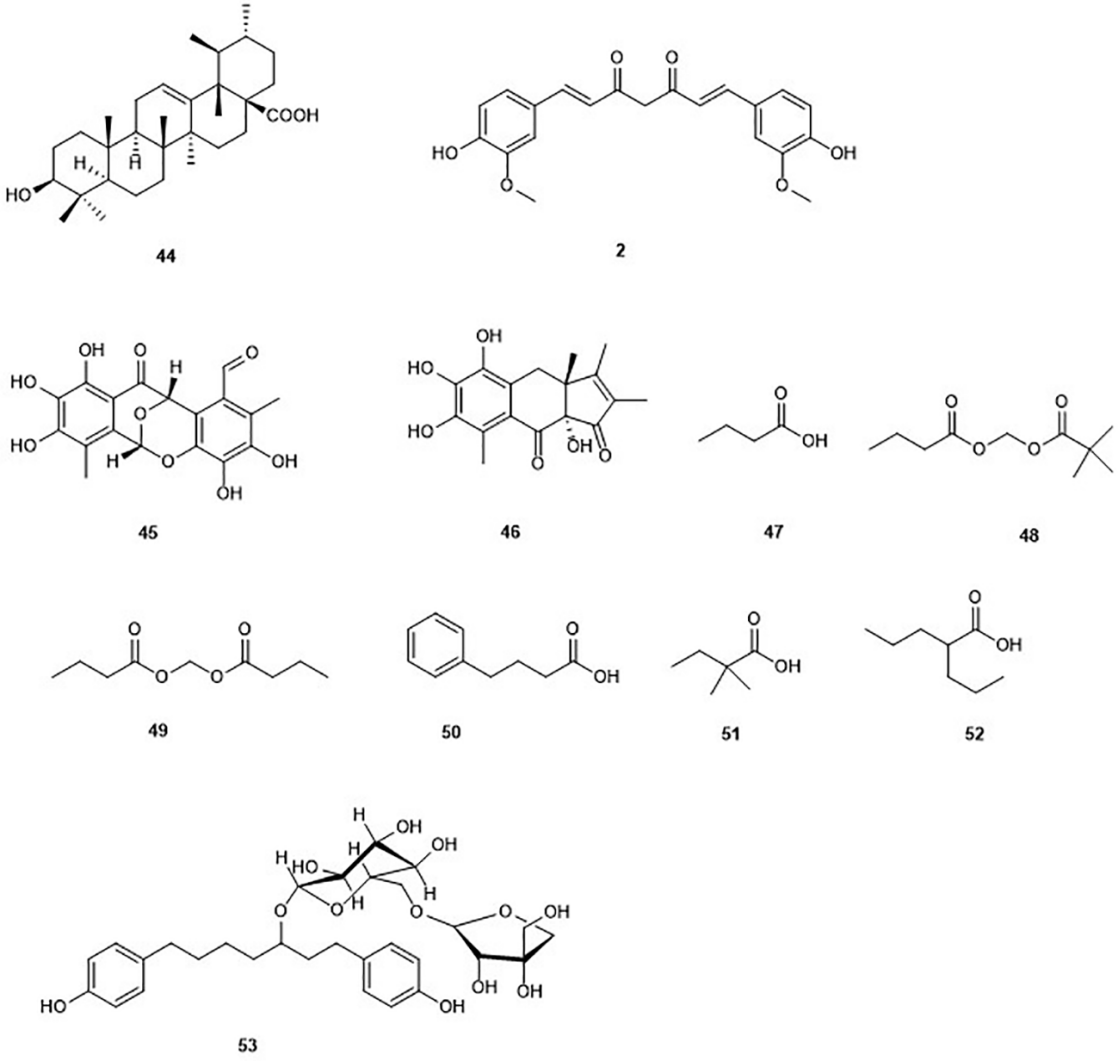
Structures of the non Zinc-binding HDACs.

#### Epicocconigrones

Epicocconigrones A (**45**) and B (**46**) are polyketides isolated from an endophytic fungus identified as *Epicoccum nigrun* in 2013 (El Amrani et al., 2014). Compound **45** displayed strong inhibition of HDAC with an IC_50_ value of 9.8 µM (El Amrani et al., 2014). *In vitro* test of **45** showed that this compound inhibits several HDACs (1, 2, 3, 8, 6, 10, and 11) with IC_50_ values between 1.6 and 12.9 µM. It is worth to mention that **45**, selectively inhibited HDAC8 with higher potency in comparison to the reference compound SAHA in the same study. Compound **45** also inhibited proliferation of the human lymphoma cell line RAJI by 50% at a concentration of 5 µM after 72 h of treatment. Moreover, 32% of the U937 cell line were also inhibited at the same concentration (El Amrani et al., 2014).

#### Curcumin

The polyphenol curcumin (**2**), as mentioned earlier, was isolated from the rhizome *Curcuma longa* (Soleimani et al., 2018). Curcumin (**2**) is known to possess HDAC inhibitory activity in different cancer cell lines (Soflaei et al., 2018). Indeed, induction by curcumin of cell cycle arrest at G2/M phase, apoptosis, and increase in tubulin acetylation in medulloblastoma cells, through the inhibition of HDAC, in particular HDAC4 was reported (Lee et al., 2011). It has been reported that **2** may be of considerable value in synergistic therapy of cancer in a manner that the drug dose level could be strongly minimized to reduce the associated toxicity (Roy et al., 2011). Indeed, **2** combined with other HDAC inhibitors such as vorinostat resulted in a marked enhancement of the antiproliferative activity of the associated drug, and sensitization to apoptosis (Giommarelli et al., 2010). A similar observation was made when **2** was combined with cyclophosphamide and paclitaxel (Roy et al., 2011). Nevertheless, curcumin is known to show pan-activities, which is why any reported specificity needs to be seen with caution.

#### *n*-Butyric Acid

*n*-Butyric acid (**47**) is a short-chain fatty acid reported as metabolite of *Staphylococcus epidermidis*, a skin probiotic bacterium (Claudel et al., 2019; Traisaeng et al., 2019). *n*-butyric acid has been reported to inhibit HDAC, DNA synthesis, and cell growth in colon tumor cell lines (Andriamihaja et al., 2009; Zhang et al., 2010; Traisaeng et al., 2019). Its poor pharmacological properties are due to a rapid metabolism. Along with the multigram doses required to achieve therapeutic concentrations *in vivo*, they precluded its use in cancer therapy and other medical disorder (Miller et al., 1987; Steliou et al., 2012). To overcome this limitation, butyric acid prodrugs have been synthesized including pivaloylomethyl butyrate (AN-9) (**48**) and butyroyloxymethyl butyrate (AN-1) (**49**), which showed antineoplastic activity and radiosensitizing capacity in the treatment of malignant gliomas (Entin-Meer et al., 2005). However, these prodrugs did not succeed as viable drugs (Steliou et al., 2012). Interestingly, the arginine salt of butyrate gave successful results in clinical studies and is used for the treatment of diseases such as thalassemia and sickle-cell disease (Steliou et al., 2012). Other short-chain fatty acids such as 4-phenylbutyrate (**50**), 2,2-dimethlbutyric acid (**51**), and valproic acid (**52**) have been synthesized and also displayed inhibition of HDAC (Steliou et al., 2012). Valproic acid (**52**) is currently used in the treatment of epilepsy (Georgoff et al., 2018). Thus, butyrate-based epigenetic compounds represent a promising route for the development of new HDADi.

#### Aceroside VIII

Aceroside VIII (**53**) is a diarylheptanoid isolated from the Japanese white birch *Betula platyphylla*. This compound weakly but selectively inhibited the activity of HDAC6 in HT29 CRC cells (Ryu et al., 2015). However, combination of this compound with the well-known selective HDAC6 inhibitor γ-lactame A452 led to a significant increase of the levels of acetylated α-tubulin. Furthermore, the treatment of HT29 CRC cells with 10 µM aceroside VIII associated with 0.1 µM A452 led to a significant decrease of cell growth up to 84% (Ryu et al., 2015). The same study showed that cell death caused by aceroside is partly dependent on caspase activation. This study highlighted a synergistic effect of natural products and selective HDAC6 inhibitors.

## Molecular Modeling and Docking as a Tool for a Mode of Action Prediction of Natural Product DNMTs and HDACs Inhibitors

Molecular docking is a computational method employed for understanding, the interaction between a small molecule (e.g. a potential drug) and its macromolecular target, e.g. a protein or receptor. Docking simulations are often used to elucidate the key binding interactions and binding modes of small molecules and their drug targets (Kellenberger et al., 2008). Scoring functions are mathematical/statistical methods implemented in docking algorithms for quantifying the interactions, hence the putative binding of a drug molecule to its target (Chen, 2015). When properly trained, a scoring function could be used as a criterion for selecting a subset of (best-scoring) ligands or small molecules, which have been stored in electronic databases (often several thousands or even millions). During a virtual screening experiment via docking, a large electronic database of ligands is docked into the binding site of a protein and putative binding is characterized using a scoring method. For each ligand, several conformers are stored as "docking poses" and the top-scoring poses are chosen as potential binders. This selected subset of compounds (called docking "hits") are then tested biologically. Thus, the number of compounds to be tested is drastically reduced, hence cutting down the cost of identification of a lead compound.

Structure-based molecular modeling (e.g. molecular docking, molecular dynamics, structure-based quantitative structure-activity relations) and ligand-based modeling (e.g. pharmacophore modeling, similarity searching) have assisted the identification of novel natural product inhibitors and modulators of DNMTs and HDACs and in explaining their inhibitory effect. Some of these results have been summarized in recent reviews (Medina-Franco and Caulfield, 2011; Saldívar-González et al., 2018).

### Interactions of Natural Compounds Within the Binding Site of DNMT1

The anthraquinone derivative Nanaomycin A (**6**), from the National Cancer Institute (NCI)/Developmental Therapeutics Program Open Chemical Repository screening program (http://dtp.cancer.gov), displayed potent antiproliferative effects on HCT116, A549, and HL60 cell lines as mentioned earlier in this review (Kuck et al., 2010). A study on the identification of DNMT1 inhibitors through a virtual screening showed that **6** induces antiproliferative effects in three different tumor cell lines (Chen et al., 2007). Furthermore, biochemical *in vitro* assay using DNMT1 or DNMT3b showed that nanaomycin A selectively inhibits DNMT3b. A docking study of nanaomycin A towards a homology model of the catalytic site of DNMT3b was conducted in order to rationalize the biochemical activity at the molecular level (Kuck et al., 2010). Indeed, docking studies confirmed that nanaomycin A binds in the active site of DNMT3b in which its carboxylic acid is capable of forming hydrogen bonds with the side chain of arginine residues. Moreover, its carbonyl oxygen atom and adjacent hydroxyl group were predicted to form an extensive hydrogen bond network with the side chain of two arginine residues. Besides, the side chain of a glutamic acid residue forms a hydrogen bond with the hydroxyl. Furthermore, a possible explanation of the selectivity of nanaomycin A for DNMT3b was suggested. Indeed, docking studies of nanaomycin A with a previously validated homology model for the catalytic site of human DNMT1 did not shown similar H-bonds with the equivalent glutamic acid and arginine residues (Siedlecki et al., 2003). Thus, the anticancer effects of nanaomycin A could be attributed to its ability to selectively inhibit DNMT3b. Thus, the anthracycline group of nanaomycin A represents a valuable scaffold for the development of future selective DNMT isoform inhibitors. However, some lasting cardiotoxicity may prevent its clinical use. It is worth to mention that nanaomycin A is the first non-SAH (*S*-adenosylhomocysteine) DNMT3b-selective compound.

**Figure 7:**
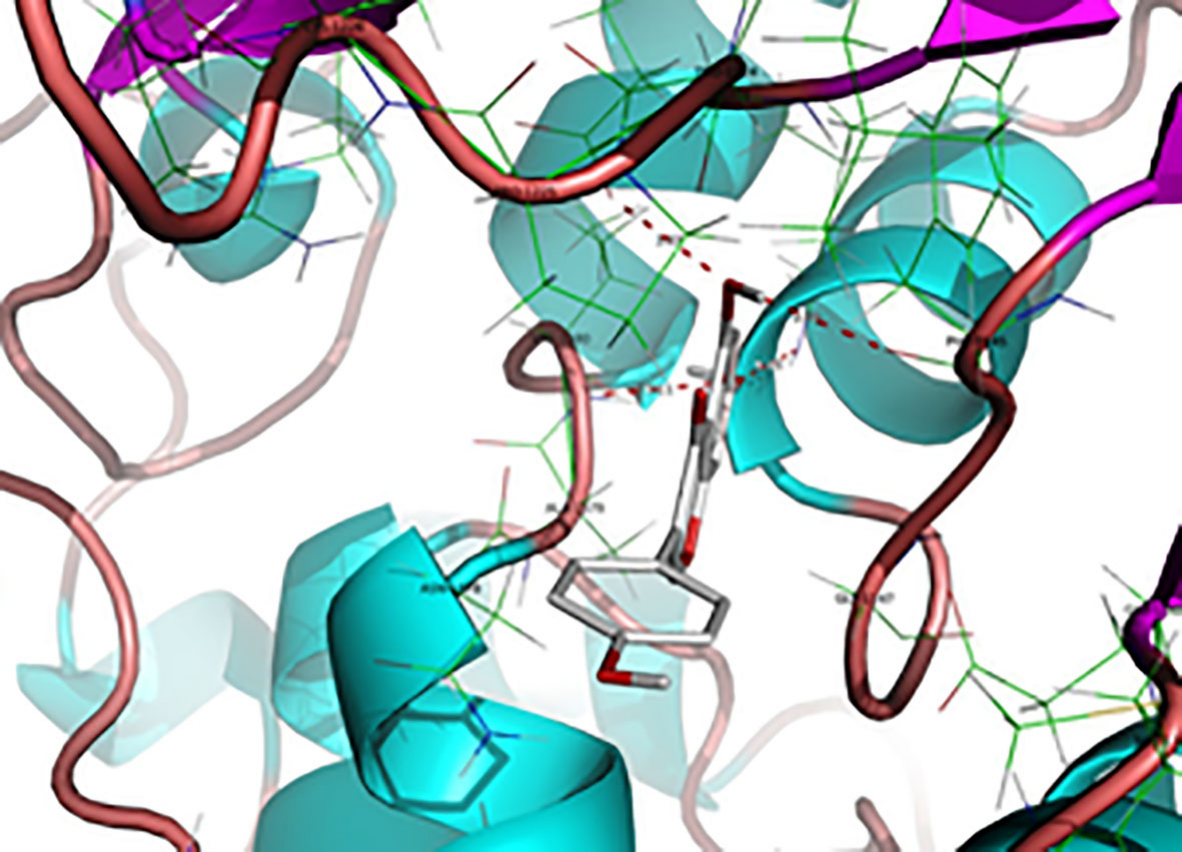
H-bond interactions between Genistein and binding site amino acid residues in the DNMT1 cavity. H-bond between genistein and DNMT1 are shown (distance <3.2 Å) as red dotted lines that include the names of the residues and distances (Reproduced with permission).

Based on the anticancer activity of the isoflavone genistein toward MDA-MB-231 human breast cancer cells and MCF-7, associated with the resulting decrease in the level of global methylation, a docking study, similar to the aforementioned nanaomycin A, was performed. Indeed, molecular modeling of the interaction between genistein and the DNMT1 binding site, as shown in **Figure 7**, revealed potential H-bond interactions (Xie et al., 2014). This study demonstrated that genistein might inhibit the binding of hemimethylated DNA, competitively to the catalytic domain of DNMT1. Moreover, the authors also demonstrated that genistein has a demetylation effect in the region of multiple tumor suppressor genes (TSG) including Adenomatous polyposis coli (APC), ataxia telangiectasia mutated (ATM), phosphatase and tensin homolog (PTEN), and increased the mRNA expression of these genes. It is worth to mention that, silencing of the expression of TSGs in cancer cells is mainly due to hypermethylation of CpG islands in the promoter region (Xie et al., 2014). These results suggested that genistein could increase the expression of certain TSGs in human breast cancer cells by reducing the activity of DNMTs and mRNA expression of DNMT1. Thus, genistein or its structural analogs could be potentially used as demethylation agents.

**Figure 8:**
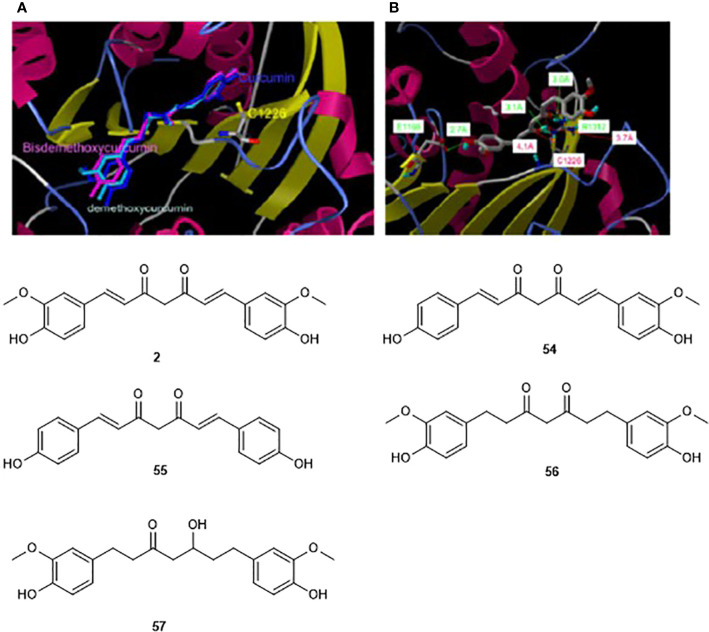
Modeling docking poses towards the homology model of showing the interaction of curcumin (2) and its two analogs (50–51) **(A)** and tetrahydrocurcumin (52, **B**) within the catalytic domain of DNMT1. The catalytic Cys1126, and anchoring Glu1668, Arg1312 are shown in the catalytic domain, (Reproduced with permission).

Similarly, molecular docking has been used to illustrate why the phenolic derivative curcumin (**2**) inhibits the enzymatic activity of an analogue of DNMT1 (M. SssI) at the lower nM range, meanwhile a close natural analogue, hexahydrocurcumin (**54**), showed no inhibition of the same enzyme up to 100 μM (Liu et al., 2009b). This finding could be explained by docking of curcumin and its tetrahydro analogue towards the catalytic domain of DNMT1. Since it was suggested experimentally that curcumin blocks the catalytic thiolate of Cys1226 of DNMT1 covalently to exert its inhibitory effect, docking validation showed the absence of such interactions in docking pose with the other docked curcumin analogues, including demethoxycurcumin (**55**), bisdemethoxycurcumin (**56**), tetrahydrocurcumin (**57**) (**Figure 8**, Liu et al., 2009b). Compounds **2**, **55**, and **56** showed similar inhibitory effects, indicating that either of bis-α,β-unsaturated ketones is required for the observation of the activity. However, as mentioned earlier, the bioactivity of curcumin should be analyzed with care.

### Discovery of Novel DNMT Inhibitors by Docking-Based Virtual Screening

Several isoforms of DNMTs occur in mammals, e.g. DNMT1, DNMT3a, and DNMT3b as mentioned earlier in this review (Chen et al., 2014). These are attractive targets in cancer chemotherapy and several crystal structures are available in the protein databank (Berman et al., 2000) for structure-based virtual screening projects (e.g. PDB ID: 4DA4) (Song et al., 2012). Several successful virtual screening campaigns *via* docking have been conducted for the discovery of DNA methyltransferase inhibitors (Kuck et al., 2010; Medina-Franco et al., 2011; Medina-Franco and Yoo, 2013; Chen et al., 2014). These include, for example, 44 natural germacrolides docked against the homology model of the human DNMT1 (Liu et al., 2009b), and the lead-like subset of ~89,425 natural products from the ZINC database (Irwin and Shoichet, 2005) which were docked against the homology model of the human DNMT1 (Medina-Franco et al., 2011). Indeed, from the docking-based screening performed by Liu and co-workers, it was observed that γ-methylactone compounds could be effective DNMT inhibitors. Moreover, the same study resulted in the discovery of parthenolide and curcumin mentioned earlier in this review. This result was confirmed by Yoo and coworkers who demonstrated that the binding models of compounds such as curcumin and parthenolide suggest that these natural products are covalent blockers of the catalytic site of DNMT (Yoo and Medina-Franco, 2011). Thus, compounds such as parthenolide are potential blockers of DNMT1.

### Natural Product HDAC Inhibitor in the PDB

Based on the evidence of HDAC inhibitory effects along with tumor-suppressing activities of SFN (**8**), Myzak et al. (2004) carried out a molecular modeling study of the SFN-Cys HDAC binding site on the HDAC crystal structure. It was shown that the buried cysteine amino group is positioned to make a single H-bond with His132 when **8** was made to interact with this binding site (Myzak et al., 2004). The same study reported that a combination of SFN (**8**) with trichostain A (**13**) led to an increase in the inhibition of the HDAC activity. It is worth to mention that **8** is metabolized into its major active form sulforaphane-cystein. Indeed, studies with SFN and media treated from SFN-treated cells indicated that the parent compound was not responsible for the HDAC inhibition activity, and this was proven through the use of glutathione S-transferase that blocked the first step in the metabolism of SFN (Myzak et al., 2004). Therefore, SFN may be an effective stand alone chemotherapeutic agent or work in synergy with other HDAC inhibitors. However, the lack of isoform selectivity inhibition may limit its potential clinical use.

Based on the known inhibitory activity of trichostatin A (TSA) (**13**), Finnin et al. (1999) carried out a molecular modeling study of this compound to establish the mechanism of HDAC inhibition (Finnin et al., 1999). This study showed that the binding of TSA proceeds by the insertion of its long aliphatic chain into the HDLP pocket, thus making multiple tube-like contacts to the hydrophobic portion of the pocket. Furthermore, it was revealed that the hydroxamic acid group of TSA coordinates the active-site zinc in a bidentate fashion using its carbonyl and hydroxyl group. It is worth to mention that other natural HDAC inhibitors such as HC-toxin (**29**) and trapoxin (**34**) mentioned earlier in this review, contain groups that are analogous to the active-site/zinc-binding groups and the cap aliphatic chain of TSA. However, they have an epoxyketone group instead of a hydroxamic acid group. It has been suggested that the epoxy group may crosslink to an active site nucleophile (Yoshida et al., 1995). Furthermore, interaction of their ketone group with polar residues and possibly the zinc, at the bottom of the active-site pocket might be possible (Finnin et al., 1999). Indeed, the reduction of the carbonyl to a hydroxyl group, or its elimination, that led to a high decrease in the activity of **29**, supported this assumption (Shute et al., 1987). This mode of action could be assumed to be identical for other HDAC inhibitors including chlamydocin (**32**), 1-alaninechlamydocin (**32**). Besides, the authors suggested that the larger size of the macrocycle (cap group) of **34** and **29** compared to that of **13** could allow more extensive contacts at the rim of the pocket and in the shallow grooves surrounding the pocket entrance.

### HDAC Inhibitors Inspired From the Natural Product Psammaplin A (PsA)

Inspired by the NP PsA (**15**), Baud et al. (2013) designed a new set of picolinamide-based histone deacetylase inhibitors, i.e. designing a focused library, which is based on the PsA core (Baud et al., 2013). Based on the HDAC inhibitory and anti-tumor (García et al., 2011) activities of this marine metabolite, the authors proceeded by probing the features of this molecule, which are responsible for its activity (Baud et al., 2012). In searching for a molecular replacement for the oxime unit of psammaplin A, Baud and coworkers were able to discover a new set easily synthesizable, isoform-selective, fragment-sized, and highly ligand efficient *N*-2-(thioethyl)picolinamide HDAC inhibitors bearing a chloropyridine motif, with low-nanomolar potencies (Baud et al., 2013). The synthesized compounds selectively inhibited HDAC1 with low-nanomolar potencies. Because selective HDAC1 inhibition has been suggested to be an effective anticancer strategy, this study showed that compounds with the chloropyridine motif will be a valuable design criterion for lead compound development of new and chemical probes that target HDAC1.

**Figure 9:**
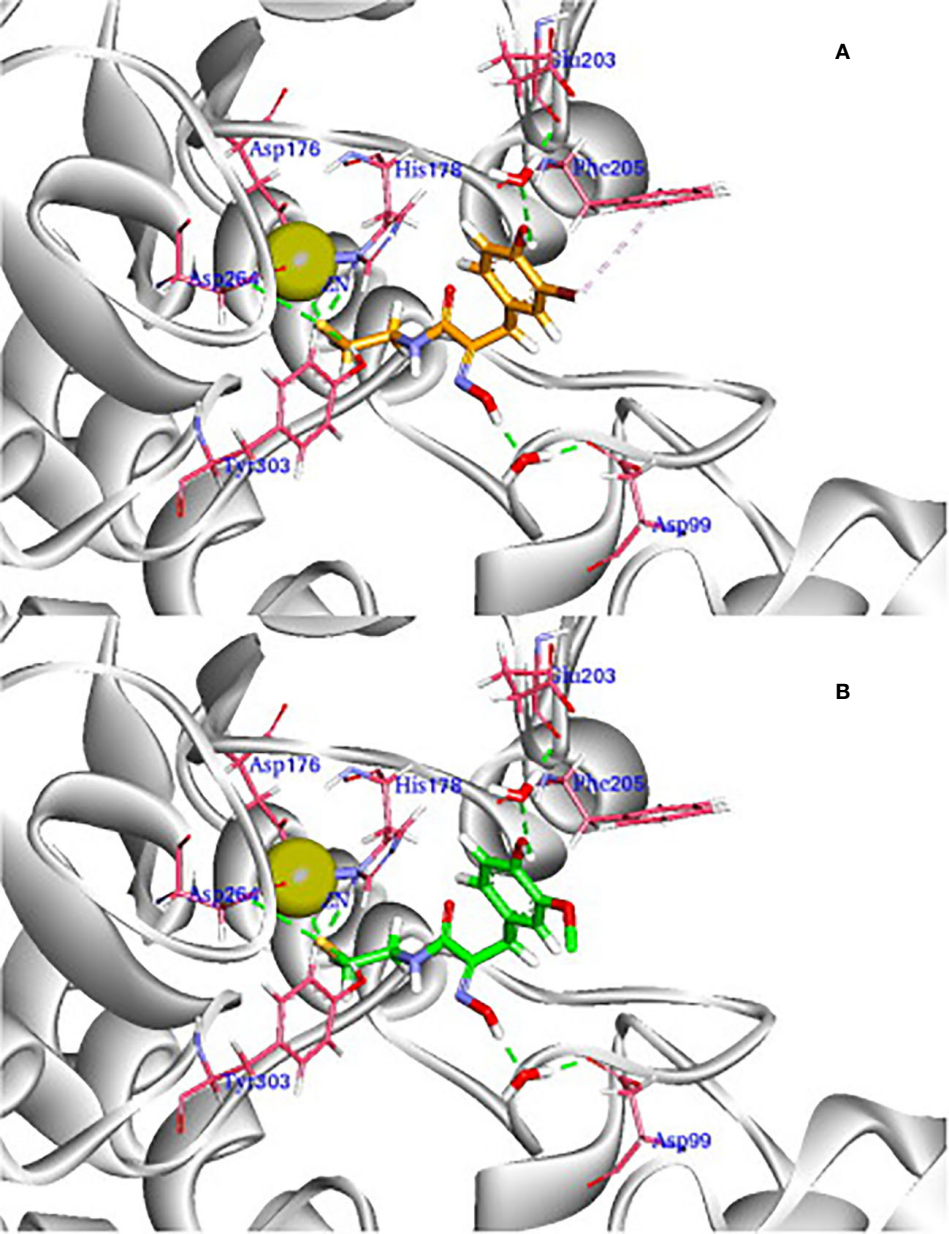
3D view of the docking pose of reduced PsA **(A)** and its synthetic analogue **(B)** to HDAC1 (PDB ID: 4BKX). The ligands are shown in orange and green, respectively. Important parts of the enzyme for interaction are shown in magenta sticks, while Zn^2+^ ion is shown as a light-yellow sphere (Reproduced with permission).

Further molecular modeling of the compound PsA and its most potent designed analogs as cytotoxic agent that act by histone deacetylase inhibition (Wen et al., 2016) was conducted against the HDAC1 binding site (PDB ID: 4BKX) (Millard et al., 2013). A comparison of the binding interactions was carried out, for example, of the synthetic analogue; (2*E*,2′*E*)-*N*,*N*′- (disulfanediyl*bis*(ethane-2,1-diyl))*bis*(2-(hydroxyimino)-3-(2,4-dichlorophenyl)propanamide, which showed better HDAC inhibitory activity than PsA and comparable antiproliferative activity with psammaplin A (**15**) against all four tested cancer cells (Wen et al., 2016). **Figure 9** shows that psammaplin A binds to HDAC1, forming key interactions with the protein in several areas. For example, the thiol group in PsA chelates the Zn^2+^ ion, while the oxime group forms an H-bond with Asp99 bridged by a water molecule. On the other hand, the 3-bromo-4-hydroxy phenyl group in PsA forms a few hydrophobic contacts to His178, Tyr204, and Phe205 around the surface recognition motif, while the hydroxyl group is optically attached to the *para*-position of benzene. This enhances interaction with Glu203 at the entrance to the active site tunnel (**Figure 9**). Besides, it was observed from molecular dynamics studies that the non-covalent interactions between the inhibitor and target protein were quite stable through 2.5 ns. The more potent synthetic analogue of this NP (shown in **Figure 9B**) shared a similar binding mode as PsA, with similar and stable interactions. However, as mentioned earlier, the physiologic instability of PsA has precluded further clinical investigations.

## Conclusion

Despite intensive research efforts, cancer is yet one of the primary global causes of death. Although numerous potent anti-cancer drugs have been developed in recent decades, there is still a huge need for specific agents with low side effects (Bray et al., 2018). DNA methylation and histone acetylation are important physiological mechanisms that maintain genome integrity. Altered DNA methylation and/or histone acetylation patterns have consistently been documented as the earliest molecular changes occurring upon tumor establishment. Since the presence of HDACs are important in both the development and progression of cancer, addressing the related epigenetic processes with HDAC inhibitors (HDACi) is a promising starting point for developing new and potent anticancer drugs (Li and Seto, 2016). HDAC inhibitors have already proven their fundamental efficacy against cancer in preclinical and clinical studies, which has led to the FDA approval of vorinostat (SAHA), romidepsin (FK288), panobinostat (LBH589), and belinostat (PXD101) for cancer therapy (Nguyen et al., 2013; Suraweera et al., 2018; Mehndiratta et al., 2020). Besides, a double-digit number of other HDAC inhibitors are currently under investigation in Phase II or III trials (Suraweera et al., 2018). Nevertheless, it should be noted that many HDAC inhibitors fail in clinical development due to lack of efficacy or too many side effects (Slingerland et al., 2014). Another major drawback is the fact that the majority of the already established HDAC inhibitors are only approved for the treatment of T-cell lymphomas, due to insufficient efficacy against solid tumors (Slingerland et al., 2014). Consequently, the identification of new HDAC inhibitors that are both potent and specific is of great importance to adequately address the pathophysiological importance of epigenetic mechanisms in modern cancer therapy. Many compounds are made chemically in academia and industry for the development of new HDACi. However, nature also offers an almost inexhaustible pool of new bioactive substances, often exhibiting novel and unexpected chemical scaffolds (Herrmann et al., 2017; Chen et al., 2018). Countless drugs based on natural products are impressive proof of the healing power hidden in nature (Dias et al., 2012). For these reasons, it seems highly advisable to screen newly discovered natural products for HDAC inhibitory activity to identify new and superior lead compounds for the development of a new generation of HDAC inhibitors. Moreover, emerging reports show that intelligently designed combination therapies with common cytostatic drugs can synergistically increase the efficacy of the inhibitors (Suraweera et al., 2018).

Many of the points discussed for HDACi apply in a similar or even the same way to DNA methyltransferase inhibitors (DNMTIs). For example, DNMTIs are most effective in hematological diseases such as myelodysplastic syndromes, whereas treatment success in solid tumors is limited (Gurion et al., 2010). Similar to HDACi, the aim is therefore to use wisely designed combination treatments to synergistically enhance the effect of well-established anti-cancer drugs through HDACi (Gnyszka et al., 2013). This approach is certainly functional, as shown by the combination with oxaliplatin or doxorubicin (Flis et al., 2009; Vijayaraghavalu et al., 2013). One problem associated with the clinical use of classical DNMTIs is that they often exhibit mutagenic effects, such as 5-azacytidine or zebularine (Amacher and Turner, 1987; Lee et al., 2004). Here, natural product-based DNMTIs such as epigallocatechin-3-gallate appear to be more advantageous, since the latter is not incorporated into the DNA, but binds directly to the catalytic region of DNMTs (Fang et al., 2003).

Natural products endowed with DNMTs and HDACs inhibition functions were reviewed in this article. Indeed, a wide range of natural compounds from plants, microorganisms, and marine sponges was presented and their potential to inhibit DNMTs and HDACs discussed. Furthermore, this study showed that compounds possessing a strong zinc-binding group are more promising HDAC inhibitors even though the presence of the latter is not a prerequisite for HDAC inhibition. Molecular modeling and docking increasingly shows to be a powerful tool for studying the interactions between the drug target and its potential inhibitors, paving the way towards the further development of novel HDAC and DNMT inhibitors as anti-tumor agents which could be natural product-inspired. However, most of the natural products presented showed an indirect effect and lack isoform selectivity, which may limit their development into clinical use. The capability to selectively inhibit single HDAC or DNMT isoforms currently represents a major challenge in the design of HDAC and DNMT inhibitors. This approach could represent an opportunity to derive improved agents which could target specific types of cancer. It is worth to mention that, combination of HDACs or DNMTs inhibitors with anti-EZH2 could increase their efficacy without overlapping toxicity (Shahabipour et al., 2017). This implies the identification of natural products that could target other epigenetic regulatory enzymes which is beyond the scope of this review.

HDACi are important for innate defense function, macrophage differentiation, and polarization (Han and Lee, 2009). Indeed, treatment of B16/F10 murine melanoma cells by the natural HDACi romidepsin showed that HDACi are promising agents in the human melanoma immunotherapy pretreatment (Murakami et al., 2008). A study by Cabanel et al. (2015) showed the importance of TsA in the regulation of macrophage differentiation and elongation (Cabanel et al., 2015). Another study also highlighted that TsA treatment inhibits inflammatory cytokine secretion and improves both CD1d and class II MHC-mediated antigen presentation. Thus, this treatment may enhance the suppression of antitumor NK T cell responses (Tiper and Webb, 2016). The same study showed that the restoration of antitumor responses to mantle cell lymphoma could be improved by treatment with HDACi. Based on these observations and despite the need of additional preclinical data to access the efficacy and toxicity of HDACi, several clinical investigations have started by associating HDACi with immunotherapeutics for patients with advanced prostate, renal, or urothelial cell carcinoma (Mazzone et al., 2017). Another study suggested that SAHA might improve the activity of the immunotherapeutic avelumab in both tumor and NK cells (Hicks et al., 2018). It is worth to mention that SAHA possesses also a hydroxamic acid function such as trichostatin A indicating that a possible derivatization of the latter could improve its efficacy. Thus, these studies suggest that combination of natural HDAC inhibitors with immunotherapeutics could improve the treatment of cancer. However, preclinical studies to access the efficacy and toxicity of this combination are still needed. A particular interest should be given on class I-specific HDACi, which are believed to provide a promising future in the cancer treatment (Mazzone et al., 2017). Indeed, combination of natural selective HDAC inhibitors might increase the anticancer drug efficacy as demonstrated in the case of the natural HDAC6-selective inhibitor aceroside VIII (Ryu et al., 2015).

However, the multitarget effects of natural products is a serious limitation of their use in the area of epigenetic drugs. Thus, chemical derivatization and molecular studies could improve their effects for a better understanding of their mechanism of action. On the other hand, the multi-target property of natural products could be utilized for the treatment of diseases including cancer, Alzheimer’s disease, and diabetic cardiomyopathy. Indeed, promising multi-target molecules have been studied for the aforementioned diseases (Musso et al., 2015; Badal et al., 2017; Karuppagounder et al., 2017). In addition, only few virtual screenings studies have been performed yet and their increase will provide hope for the discovery of potential DNMT and HDAC inhibitors. Nonetheless, the structures presented in this review offer the well-founded basis that screening and chemical modifications of natural products will in future provide not only leads to the identification of more specific inhibitors with fewer side effects, but also important features for the elucidation of HDAC and DNMT function for cancer treatment.

## Author Contributions

SA and RM edited and reviewed the manuscript. SA wrote the first draft of the manuscript. SA, FN-K, FS, ME, AN, and SM wrote sections of the manuscript. All authors contributed to the article and approved the submitted version.

## Conflict of Interest

The authors declare that the research was conducted in the absence of any commercial or financial relationships that could be construed as a potential conflict of interest.

## References

[B1] Ahn M. Y. Jung J. H. Na Y. J. Kim H. S. (2008). A natural histone deacetylase inhibitor, Psammaplin A, induces cell cycle arrest and apoptosis in human endometrial cancer cells. Gynecol. Oncol. 108, 27–33. 10.1016/j.ygyno.2007.08.098 17920664

[B2] Ahn M. Y. Kang O. D. Na J. Y. Yoon S. Choi S. W. Kang W. K. (2012). Histone deacetylase inhibitor, apicidin, inhibits human ovarian cancer cell migration via class II histone deacetylase 4 silencing. Cancer Lett. 325, 189–199. 10.1016/j.canlet.2012.06.017 22781396

[B3] Akihisa T. Yasukawa K. Tokuda H. (2003). “Potentially Cancer Chemopreventive And Anti-Inflammatory Terpenoids From Natural Sources,” in Studies in Natural Products Chemistry : Bioactive Natural Products (Part J). Ed. Atta-ur-Rahman (Elsevier), 73–126.

[B4] Akiyama T. Ishida J. Nakagawa S. Ogawara H. Watanabe S. Itoh N. (1987). Genistein, a specific inhibitor of tyrosine-specific protein kinases. J. Biol. Chem. 262, 5592–5595. 3106339

[B5] Allis C. D. Jenuwein T. (2016). The molecular hallmarks of epigenetic control. Nat. Rev. Genet. 17, 487–500. 10.1038/nrg.2016.59 27346641

[B6] Alvarez M. C. Maso V. Torello C. O. Ferro K. P. Saad S. T. O. (2018). The polyphenol quercetin induces cell death in leukemia by targeting epigenetic regulators of pro-apoptotic genes. Clin. Epigenet. 10, 1–11. 10.1186/s13148-018-0563-3 PMC6225654 30409182

[B7] Amacher D. E. Turner G. N. (1987). The mutagenicity of 5-azacytidine and other inhibitors of replicative DNA synthesis in the L5178Y mouse lymphoma cell. Mutat. Res./Fundam. Mol. Mech. Mutagenesis 176, 123–131. 10.1016/0027-5107(87)90259-4 2432424

[B8] Andriamihaja M. Chaumontet C. Tome D. Blachier F. (2009). Butyrate metabolism in human colon carcinoma cells: implications concerning its growth-inhibitory effect. J. Cell. Physiol. 218, 58–65. 10.1002/jcp.21556 18767040

[B9] Auclair G. Weber M. (2012). Mechanisms of DNA methylation and demethylation in mammals. Biochimie 94, 2202–2211. 10.1016/j.biochi.2012.05.016 22634371

[B10] Badal S. A. M. Aiken W. D. Chin S. N. (2017). Molecular Targets and Angiogenesis in Renal Cell Carcinoma, A Multitarget Approach: Mini Review. Curr. Drug Targets 18, 1204–1213. 10.2174/1389450117666160502152518 27138755

[B11] Bae J. Kumazoe M. Fujimura Y. Tachibana H. (2019). Diallyl disulfide potentiates anti-obesity effect of green tea in high-fat/high-sucrose diet-induced obesity. J. Nutr. Biochem. 64, 152–161. 10.1016/j.jnutbio.2018.10.014 30504007

[B12] Baud M. G. J. Leiser T. Haus P. Samlal S. Wong A. C. Wood R. J. (2012). Defining the mechanism of action and enzymatic selectivity of psammaplin A against its epigenetic targets. J. Med. Chem. 55, 1731–1750. 10.1021/jm2016182 22280363

[B13] Baud M. G. J. Haus P. Leiser T. Meyer-Almes F.-J. Fuchter M. J. (2013). Highly ligand efficient and selective N-2-(Thioethyl)picolinamide histone deacetylase inhibitors inspired by the natural product psammaplin A. ChemMedChem 8, 149–156. 10.1002/cmdc.201200450 23184734

[B14] Benelkebir H. Marie S. Hayden A. L. Lyle J. Loadman P. M. Crabb S. J. (2011). Total synthesis of largazole and analogues: HDAC inhibition, antiproliferative activity and metabolic stability. Bioorg. Med. Chem. 19, 3650–3658. 10.1016/j.bmc.2011.02.024 21420302

[B15] Berman H. M. Westbrook J. Feng Z. Gilliland G. Bhat T. N. Weissig H. (2000). The Protein Data Bank. Nucleic Acids Res. 28, 235–242. 10.1093/nar/28.1.235 10592235 PMC102472

[B16] Bi Y. Min M. Shen W. Liu Y. (2018). Genistein induced anticancer effects on pancreatic cancer cell lines involves mitochondrial apoptosis, G0/G1cell cycle arrest and regulation of STAT3 signalling pathway. Phytomedicine 39, 10–16. 10.1016/j.phymed.2017.12.001 29433670

[B17] Bougdour A. Maubon D. Baldacci P. Ortet P. Bastien O. Bouillon A. (2009). Drug inhibition of HDAC3 and epigenetic control of differentiation in Apicomplexa parasites. J. Exp. Med. 206, 953–966. 10.1084/jem.20082826 19349466 PMC2715132

[B18] Bray F. Ferlay J. Soerjomataram I. Siegel R. L. Torre L. A. Jemal A. (2018). Global cancer statistics 2018: GLOBOCAN estimates of incidence and mortality worldwide for 36 cancers in 185 countries. CA Cancer J. Clin. 68, 394–424. 10.3322/caac.21492 30207593

[B19] Cabanel M. Brand C. Oliveira-Nunes M. C. Cabral-Piccin M. P. Lopes M. F. Brito J. M. (2015). Epigenetic Control of Macrophage Shape Transition towards an Atypical Elongated Phenotype by Histone Deacetylase Activity. PloS One 10, e0132984. 10.1371/journal.pone.0132984 26196676 PMC4509762

[B20] Cabrera C. Artacho R. Giménez R. (2006). Beneficial effects of green tea–a review. J. Am. Coll. Nutr. 25, 79–99. 10.1080/07315724.2006.10719518 16582024

[B21] Cal C. Garban H. Jazirehi A. Yeh C. Mizutani Y. Bonavida B. (2003). Resveratrol and cancer: chemoprevention, apoptosis, and chemo-immunosensitizing activities. Curr. Med. Chem. Anticancer Agents 3, 77–93. 10.2174/1568011033353443 12678904

[B22] Caulfield T. Medina-Franco J. L. (2011). Molecular dynamics simulations of human DNA methyltransferase 3B with selective inhibitor nanaomycin A. J. Struct. Biol. 176, 185–191. 10.1016/j.jsb.2011.07.015 21839172

[B23] Chen T. Hevi S. Gay F. Tsujimoto N. He T. Zhang B. (2007). Complete inactivation of DNMT1 leads to mitotic catastrophe in human cancer cells. Nat. Genet. 39, 391–396. 10.1038/ng1982 17322882

[B24] Chen I.-H. Lu M.-C. Du Y.-C. Yen M.-H. Wu C.-C. Chen Y.-H. (2009). Cytotoxic triterpenoids from the stems of Microtropis japonica. J. Nat. Prod. 72, 1231–1236. 10.1021/np800694b 19534471

[B25] Chen S. Wang Y. Zhou W. Li S. Peng J. Shi Z. (2014). Identifying novel selective non-nucleoside DNA methyltransferase 1 inhibitors through docking-based virtual screening. J. Med. Chem. 57, 9028–9041. 10.1021/jm501134e 25333769

[B26] Chen Y. Garcia de Lomana M. Friedrich N.-O. Kirchmair J. (2018). Characterization of the Chemical Space of Known and Readily Obtainable Natural Products. J. Chem. Inf. Model 58, 1518–1532. 10.1021/acs.jcim.8b00302 30010333

[B27] Chen J. Ying Y. Zhu H. Zhu T. Qu C. Jiang J. (2019a). Curcumin-induced promoter hypermethylation of the mammalian target of rapamycin gene in multiple myeloma cells. Oncol. Lett. 17, 1108–1114. 10.3892/ol.2018.9662 30655872 PMC6312997

[B28] Chen L. Chan L. S. Lung H. L. Yip T. T. C. Ngan R. K. C. Wong J. W. C. (2019b). Crucifera sulforaphane (SFN) inhibits the growth of nasopharyngeal carcinoma through DNA methyltransferase 1 (DNMT1)/Wnt inhibitory factor 1 (WIF1) axis. Phytomedicine 63, 153058. 10.1016/j.phymed.2019.153058 31394414

[B29] Chen Y.-C. (2015). Beware of docking! Trends Pharmacol. Sci. 36, 78–95. 25543280 10.1016/j.tips.2014.12.001

[B30] Cherblanc F. L. Davidson R. W. M. Di Fruscia P. Srimongkolpithak N. Fuchter M. J. (2013). Perspectives on natural product epigenetic modulators in chemical biology and medicine. Nat. Prod. Rep. 30, 605–624. 10.1039/c3np20097c 23396528

[B31] Choi S. Y. Kee H. J. Jin L. Ryu Y. Sun S. Kim G. R. (2018). Inhibition of class IIa histone deacetylase activity by gallic acid, sulforaphane, TMP269, and panobinostat. BioMed. Pharmacother. 101, 145–154. 10.1016/j.biopha.2018.02.071 29482060

[B32] Claudel J.-P. Auffret N. Leccia M.-T. Poli F. Corvec S. Dréno B. (2019). Staphylococcus epidermidis: A Potential New Player in the Physiopathology of Acne? Dermatol. (Basel) 235, 287–294. 10.1159/000499858 31112983

[B33] Closse A. Huguenin R. (1974). Isolierung und Strukturaufklärung von Chlamydocin. Helv. Chim. Acta 57, 533–545. 10.1002/hlca.19740570306 4857466

[B34] Colletti S. L. Myers R. W. Darkin-Rattray S. J. Gurnett A. M. Dulski P. M. Galuska S. (2001). Broad spectrum antiprotozoal agents that inhibit histone deacetylase: structure–activity relationships of apicidin. Part 1. Bioorg. Med. Chem. Lett. 11, 107–111. 10.1016/S0960-894X(00)00604-1 11206438

[B35] Cuneo K. C. Fu A. Osusky K. Huamani J. Hallahan D. E. Geng L. (2007). Histone deacetylase inhibitor NVP-LAQ824 sensitizes human nonsmall cell lung cancer to the cytotoxic effects of ionizing radiation. Anticancer Drugs 18, 793–800. 10.1097/CAD.0b013e3280b10d57 17581301

[B36] D’Acunto C. W. Fontanella B. Rodriquez M. Taddei M. Parente L. Petrella A. (2010). Histone deacetylase inhibitor FR235222 sensitizes human prostate adenocarcinoma cells to apoptosis through up-regulation of Annexin A1. Cancer Lett. 295, 85–91. 10.1016/j.canlet.2010.02.016 20227822

[B37] Das P. M. Singal R. (2004). DNA methylation and cancer. J. Clin. Oncol. 22, 4632–4642. 10.1200/JCO.2004.07.151 15542813

[B38] Dawson M. A. (2017). The cancer epigenome: Concepts, challenges, and therapeutic opportunities. Science 355, 1147–1152. 10.1126/science.aam7304 28302822

[B39] Deng X. Qiu Q. He K. Cao X. (2018). The seekers: how epigenetic modifying enzymes find their hidden genomic targets in Arabidopsis. Curr. Opin. Plant Biol. 45, 75–81. 10.1016/j.pbi.2018.05.006 29864678

[B40] Deubzer H. E. Ehemann V. Westermann F. Heinrich R. Mechtersheimer G. Kulozik A. E. (2008). Histone deacetylase inhibitor Helminthosporium carbonum (HC)-toxin suppresses the malignant phenotype of neuroblastoma cells. Int. J. Cancer 122, 1891–1900. 10.1002/ijc.23295 18074352

[B41] Dias D. A. Urban S. Roessner U. (2012). A historical overview of natural products in drug discovery. Metabolites 2, 303–336. 10.3390/metabo2020303 24957513 PMC3901206

[B42] Dong L. Shi W. Li W.-H. Li Y. Sun Y.-Q. (2019). Parthenolide induces apoptosis and inhibits proliferation of human 786-O kidney cancer cells in vitro. Int. J. Clin. Exp. Med. 12, 7056–7064.

[B43] Druesne N. Pagniez A. Mayeur C. Thomas M. Cherbuy C. Duée P.-H. (2004). Diallyl disulfide (DADS) increases histone acetylation and p21(waf1/cip1) expression in human colon tumor cell lines. Carcinogenesis 25, 1227–1236. 10.1093/carcin/bgh123 14976134

[B44] Du L. Risinger A. L. King J. B. Powell D. R. Cichewicz R. H. (2014). A potent HDAC inhibitor, 1-alaninechlamydocin, from a Tolypocladium sp. induces G2/M cell cycle arrest and apoptosis in MIA PaCa-2 cells. J. Nat. Prod. 77, 1753–1757. 10.1021/np500387h 24999749 PMC4113265

[B45] Duraisingh M. T. Skillman K. M. (2018). Epigenetic Variation and Regulation in Malaria Parasites. Annu. Rev. Microbiol. 72, 355–375. 10.1146/annurev-micro-090817-062722 29927705

[B46] El Amrani M. Lai D. Debbab A. Aly A. H. Siems K. Seidel C. (2014). Protein kinase and HDAC inhibitors from the endophytic fungus Epicoccum nigrum. J. Nat. Prod. 77, 49–56. 10.1021/np4005745 24328302

[B47] Ellis L. Hammers H. Pili R. (2009). Targeting tumor angiogenesis with histone deacetylase inhibitors. Cancer Lett. 280, 145–153. 10.1016/j.canlet.2008.11.012 19111391 PMC2814368

[B48] Entin-Meer M. Rephaeli A. Yang X. Nudelman A. VandenBerg S. R. Haas-Kogan D. A. (2005). Butyric acid prodrugs are histone deacetylase inhibitors that show antineoplastic activity and radiosensitizing capacity in the treatment of malignant gliomas. Mol. Cancer Ther. 4, 1952–1961. 10.1158/1535-7163.MCT-05-0087 16373710

[B49] Esteller M. (2007). Cancer epigenomics: DNA methylomes and histone-modification maps. Nat. Rev. Genet. 8, 286–298. 10.1038/nrg2005 17339880

[B50] Esteller M. (2008). Epigenetics in cancer. N Engl. J. Med. 358, 1148–1159. 10.1056/NEJMra072067 18337604

[B51] Fahey J. W. Zhang Y. Talalay P. (1997). Broccoli sprouts: an exceptionally rich source of inducers of enzymes that protect against chemical carcinogens. Proc. Natl. Acad. Sci. U.S.A. 94, 10367–10372. 10.1073/pnas.94.19.10367 9294217 PMC23369

[B52] Fang M. Z. Wang Y. Ai N. Hou Z. Sun Y. Lu H. (2003). Tea Polyphenol (–)-Epigallocatechin-3-Gallate Inhibits DNA Methyltransferase and Reactivates Methylation-Silenced Genes in Cancer Cell Lines. Cancer Res. 63, 7563–7570. 14633667

[B53] Ferioli M. Zauli G. Maiorano P. Milani D. Mirandola P. Neri L. M. (2019). Role of physical exercise in the regulation of epigenetic mechanisms in inflammation, cancer, neurodegenerative diseases, and aging process. J. Cell. Physiol. 234, 14852–14864. 10.1002/jcp.28304 30767204

[B54] Finnin M. S. Donigian J. R. Cohen A. Richon V. M. Rifkind R. A. Marks P. A. (1999). Structures of a histone deacetylase homologue bound to the TSA and SAHA inhibitors. Nature 401, 188–193. 10.1038/43710 10490031

[B55] Fleming I. Iqbal J. Krebs E.-P. (1983). The total synthesis of (± )- trichostatin A: Some observations on the acylation and alkylation of silyl enol ethers, silyl dienol ethers and a silyl trienol ether. TETRAHEDRON 39, 841–846. 10.1016/S0040-4020(01)88581-1

[B56] Flis S. Gnyszka A. Misiewicz-Krzemińska I. Spławiński J. (2009). Decytabine enhances cytotoxicity induced by oxaliplatin and 5-fluorouracil in the colorectal cancer cell line Colo-205. Cancer Cell Int. 9, 1–10. 10.1186/1475-2867-9-10 19397792 PMC2683807

[B57] Freund R. R. A. Gobrecht P. Moser P. Fischer D. Arndt H.-D. (2019). Synthesis and biological profiling of parthenolide ether analogs. Org. Biomol. Chem. 17, 9703–9707. 10.1039/C9OB02166C 31701984

[B58] Furumai R. Komatsu Y. Nishino N. Khochbin S. Yoshida M. Horinouchi S. (2001). Potent histone deacetylase inhibitors built from trichostatin A and cyclic tetrapeptide antibiotics including trapoxin. Curr. Biol. 98, 87–92. 10.1073/pnas.98.1.87 PMC14549 11134513

[B59] Furumai R. Matsuyama A. Kobashi N. Lee K.-H. Nishiyama M. Nakajima H. (2002). FK228 (Depsipeptide) as a Natural Prodrug That Inhibits Class I Histone Deacetylases. Cancer Res. 62, 4916–4921. 12208741

[B60] Gao L. Cheng D. Yang J. Wu R. Li W. Kong A.-N. (2018). Sulforaphane epigenetically demethylates the CpG sites of the miR-9-3 promoter and reactivates miR-9-3 expression in human lung cancer A549 cells. J. Nutr. Biochem. 56, 109–115. 10.1016/j.jnutbio.2018.01.015 29525530 PMC8111486

[B61] García J. Franci G. Pereira R. Benedetti R. Nebbioso A. Rodríguez-Barrios F. (2011). Epigenetic profiling of the antitumor natural product psammaplin A and its analogues. Bioorg. Med. Chem. 19, 3637–3649. 10.1016/j.bmc.2010.12.026 21215647

[B62] Georgoff P. E. Nikolian V. C. Bonham T. Pai M. P. Tafatia C. Halaweish I. (2018). Safety and Tolerability of Intravenous Valproic Acid in Healthy Subjects: A Phase I Dose-Escalation Trial. Clin. Pharmacokinet. 57, 209–219. 10.1007/s40262-017-0553-1 28497259 PMC5699961

[B63] Ghantous A. Saikali M. Rau T. Gali-Muhtasib H. Schneider-Stock R. Darwiche N. (2012). Inhibition of tumor promotion by parthenolide: epigenetic modulation of p21. Cancer Prev. Res. (Phila) 5, 1298–1309. 10.1158/1940-6207.CAPR-12-0230 23037503

[B64] Giommarelli C. Zuco V. Favini E. Pisano C. Dal Piaz F. de Tommasi N. (2010). The enhancement of antiproliferative and proapoptotic activity of HDAC inhibitors by curcumin is mediated by Hsp90 inhibition. Cell. Mol. Life Sci. 67, 995–1004. 10.1007/s00018-009-0233-x 20039095 PMC11115870

[B65] Glozak M. A. Seto E. (2007). Histone deacetylases and cancer. Oncogene 26, 5420–5432. 10.1038/sj.onc.1210610 17694083

[B66] Gnyszka A. Jastrzebski Z. Flis S. (2013). DNA methyltransferase inhibitors and their emerging role in epigenetic therapy of cancer. Anticancer Res. 33, 2989–2996. 23898051

[B67] Gołąbek K. Strzelczyk J. K. Wiczkowski A. Michalski M. (2015). Potential use of histone deacetylase inhibitors in cancer therapy. Contemp. Oncol. (Pozn) 19, 436–440. 10.5114/wo.2015.51824 26843838 PMC4731444

[B68] Gu W. Cueto M. Jensen P. R. Fenical W. Silverman R. B. (2007). Microsporins A and B: new histone deacetylase inhibitors from the marine-derived fungus Microsporum cf. gypseum and the solid-phase synthesis of microsporin A. Tetrahedron 63, 6535–6541. 10.1016/j.tet.2007.04.025

[B69] Gurion R. Vidal L. Gafter-Gvili A. Belnik Y. Yeshurun M. Raanani P. (2010). 5-azacitidine prolongs overall survival in patients with myelodysplastic syndrome–a systematic review and meta-analysis. Haematologica 95, 303–310. 10.3324/haematol.2009.010611 19773261 PMC2817034

[B70] Haberland M. Montgomery R. L. Olson E. N. (2009). The many roles of histone deacetylases in development and physiology: implications for disease and therapy. Nat. Rev. Genet. 10, 32–42. 10.1038/nrg2485 19065135 PMC3215088

[B71] Halkidou K. Gaughan L. Cook S. Leung H. Y. Neal D. E. Robson C. N. (2004). Upregulation and nuclear recruitment of HDAC1 in hormone refractory prostate cancer. Prostate 59, 177–189. 10.1002/pros.20022 15042618

[B72] Han S.-B. Lee J. K. (2009). Anti-inflammatory effect of Trichostatin-A on murine bone marrow-derived macrophages. Arch. Pharm. Res. 32, 613–624. 10.1007/s12272-009-1418-4 19407980

[B73] Han J.-W. Ahn S. H. Park S. H. Wang S. Y. Bae G.-U. Seo D.-W. (2000). Apicidin, a Histone Deacetylase Inhibitor, Inhibits Proliferation of Tumor Cells via Induction of p21WAF1/Cip1 and Gelsolin. Cancer Res. 60, 6068–6074. 11085529

[B74] Hauser A.-T. Jung M. (2008). Targeting epigenetic mechanisms: potential of natural products in cancer chemoprevention. Planta Med. 74, 1593–1601. 10.1055/s-2008-1081347 18704881

[B75] Herman-Antosiewicz A. Singh S. V. (2004). Signal transduction pathways leading to cell cycle arrest and apoptosis induction in cancer cells by Allium vegetable-derived organosulfur compounds: a review. Mutat. Res. 555, 121–131. 10.1016/j.mrfmmm.2004.04.016 15476856

[B76] Herrmann J. Fayad A. A. Müller R. (2017). Natural products from myxobacteria: novel metabolites and bioactivities. Nat. Prod. Rep. 34, 135–160. 10.1039/C6NP00106H 27907217

[B77] Hicks K. C. Fantini M. Donahue R. N. Schwab A. Knudson K. M. Tritsch S. R. (2018). Epigenetic priming of both tumor and NK cells augments antibody-dependent cellular cytotoxicity elicited by the anti-PD-L1 antibody avelumab against multiple carcinoma cell types. Oncoimmunology 7, e1466018. 10.1080/2162402X.2018.1466018 30377559 PMC6205056

[B78] Ho E. Clarke J. D. Dashwood R. H. (2009). Dietary sulforaphane, a histone deacetylase inhibitor for cancer prevention. J. Nutr. 139, 2393–2396. 10.3945/jn.109.113332 19812222 PMC2777483

[B79] Hong J. Luesch H. (2012). Largazole: from discovery to broad-spectrum therapy. Nat. Prod. Rep. 29, 449–456. 10.1039/c2np00066k 22334030 PMC4777309

[B80] Houghton C. A. (2019). Sulforaphane: Its “Coming of Age” as a Clinically Relevant Nutraceutical in the Prevention and Treatment of Chronic Disease. Oxid. Med. Cell Longev. 2019, 2716870. 10.1155/2019/2716870 31737167 PMC6815645

[B81] Hsiang C.-H. Tunoda T. Whang Y. E. Tyson D. R. Ornstein D. K. (2006). The impact of altered annexin I protein levels on apoptosis and signal transduction pathways in prostate cancer cells. Prostate 66, 1413–1424. 10.1002/pros.20457 16741918

[B82] Huang B. H. Laban M. Leung C. H.-W. Lee L. Lee C. K. Salto-Tellez M. (2005). Inhibition of histone deacetylase 2 increases apoptosis and p21Cip1/WAF1 expression, independent of histone deacetylase 1. Cell Death Differ. 12, 395–404. 10.1038/sj.cdd.4401567 15665816

[B83] Ikeda Y. Murakami A. Ohigashi H. (2008). Ursolic acid: an anti- and pro-inflammatory triterpenoid. Mol. Nutr. Food Res. 52, 26–42. 10.1002/mnfr.200700389 18203131

[B84] Irwin J. J. Shoichet B. K. (2005). ZINC–a free database of commercially available compounds for virtual screening. J. Chem. Inf. Model 45, 177–182. 10.1021/ci049714+ 15667143 PMC1360656

[B85] Ishola A. A. Adewole K. E. (2019). Phytosterols and triterpenes from Morinda lucida Benth. exhibit binding tendency against class I HDAC and HDAC7 isoforms. Mol. Biol. Rep. 46, 2307–2325. 10.1007/s11033-019-04689-8 30771146

[B86] Itazaki H. Nagashima K. Sugita K. Yoshida H. Kawamura Y. Yasuda Y. (1990). Isolation and structural elucidation of new cyclotetrapeptides, trapoxins A and B, having detransformation activities as antitumor agents. J. Antibiot. 43, 1524–1532. 10.7164/antibiotics.43.1524 2276972

[B87] Jacob C. (2006). A scent of therapy: pharmacological implications of natural products containing redox-active sulfur atoms. Nat. Prod. Rep. 23, 851–863. 10.1039/b609523m 17119635

[B88] Jang M. Cai L. Udeani G. O. Slowing K. V. Thomas C. F. Beecher C. W. (1997). Cancer chemopreventive activity of resveratrol, a natural product derived from grapes. Science 275, 218–220. 10.1126/science.275.5297.218 8985016

[B89] Jasek K. Kubatka P. Samec M. Liskova A. Smejkal K. Vybohova D. (2019). DNA Methylation Status in Cancer Disease: Modulations by Plant-Derived Natural Compounds and Dietary Interventions. Biomolecules 9, 289. 10.3390/biom9070289 PMC6680848 31323834

[B90] Jeong J.-H. An J. Y. Kwon Y. T. Rhee J. G. Lee Y. J. (2009). Effects of low dose quercetin: cancer cell-specific inhibition of cell cycle progression. J. Cell Biochem. 106, 73–82. 10.1002/jcb.21977 19009557 PMC2736626

[B91] Jones P. A. Baylin S. B. (2007). The epigenomics of cancer. Cell 128, 683–692. 10.1016/j.cell.2007.01.029 17320506 PMC3894624

[B92] Joung K. E. Kim D.-K. Sheen Y. Y. (2004). Antiproliferative effect of trichostatin a and hc-toxin in T47D Human breast cancer cells. Arch. Pharm. Res. 27, 640–645. 10.1007/BF02980164 15283467

[B93] Kala R. Shah H. N. Martin S. L. Tollefsbol T. O. (2015). Epigenetic-based combinatorial resveratrol and pterostilbene alters DNA damage response by affecting SIRT1 and DNMT enzyme expression, including SIRT1-dependent γ-H2AX and telomerase regulation in triple-negative breast cancer. BMC Cancer 15, 1–18. 10.1186/s12885-015-1693-z 26459286 PMC4603342

[B94] Karuppagounder V. Arumugam S. Giridharan V. V. Sreedhar R. Bose R. J. C. Vanama J. (2017). Tiny molecule, big power: Multi-target approach for curcumin in diabetic cardiomyopathy. Nutrition 34, 47–54. 10.1016/j.nut.2016.09.005 28063511

[B95] Kellenberger E. Foata N. Rognan D. (2008). Ranking targets in structure-based virtual screening of three-dimensional protein libraries: methods and problems. J. Chem. Inf. Model 48, 1014–1025. 10.1021/ci800023x 18412328

[B96] Khan N. Jeffers M. Kumar S. Hackett C. Boldog F. Khramtsov N. (2008). Determination of the class and isoform selectivity of small-molecule histone deacetylase inhibitors. Biochem. J. 409, 581–589. 10.1042/BJ20070779 17868033

[B97] Khan N. Bharali D. J. Adhami V. M. Siddiqui I. A. Cui H. Shabana S. M. (2014). Oral administration of naturally occurring chitosan-based nanoformulated green tea polyphenol EGCG effectively inhibits prostate cancer cell growth in a xenograft model. Carcinogenesis 35, 415–423. 10.1093/carcin/bgt321 24072771 PMC3908746

[B98] Khan M. A. Hussain A. Sundaram M. K. Alalami U. Gunasekera D. Ramesh L. (2015). (-)-Epigallocatechin-3-gallate reverses the expression of various tumor-suppressor genes by inhibiting DNA methyltransferases and histone deacetylases in human cervical cancer cells. Oncol. Rep. 33, 1976–1984. 10.3892/or.2015.3802 25682960

[B99] Kijima M. Yoshida M. Sugita K. Horinouchi S. Beppu T. (1993). Trapoxin, an antitumor cyclic tetrapeptide, is an irreversible inhibitor of mammalian histone deacetylase. J. Biol. Chem. 268, 22429–22435. 8226751

[B100] Kim J. K. Park S. U. (2016). Current potential health benefits of sulforaphane. EXCLI J. 15, 571–577. 10.17179/excli2016-485 28096787 PMC5225737

[B101] Kim J.-S. Lee S. Lee T. Lee Y.-W. Trepel J. B. (2001). Transcriptional Activation of p21WAF1/CIP1 by Apicidin, a Novel Histone Deacetylase Inhibitor. Biochem. Biophys. Res. Commun. 281, 866–871. 10.1006/bbrc.2001.4434 11237739

[B102] Kim D. H. Shin J. Kwon H. J. (2007). Psammaplin A is a natural prodrug that inhibits class I histone deacetylase. Exp. Mol. Med. 39, 47–55. 10.1038/emm.2007.6 17334228

[B103] Klausmeyer P. Shipley S. M. Zuck K. M. McCloud T. G. (2011). Histone deacetylase inhibitors from Burkholderia thailandensis. J. Nat. Prod. 74, 2039–2044. 10.1021/np200532d 21967146 PMC3204006

[B104] Kuck D. Caulfield T. Lyko F. Medina-Franco J. L. (2010). Nanaomycin A selectively inhibits DNMT3B and reactivates silenced tumor suppressor genes in human cancer cells. Mol. Cancer Ther. 9, 3015–3023. 10.1158/1535-7163.MCT-10-0609 20833755

[B105] Kwon H. J. Owa T. Hassig C. A. Shimada J. Schreiber S. L. (1998). Depudecin induces morphological reversion of transformed fibroblasts via the inhibition of histone deacetylase. Proc. Natl. Acad. Sci. U.S.A. 95, 3356–3361. 10.1073/pnas.95.7.3356 9520369 PMC19839

[B106] Kwon H. J. Smith W. C. Xiang L. Shen B. (2001). Cloning and heterologous expression of the macrotetrolide biosynthetic gene cluster revealed a novel polyketide synthase that lacks an acyl carrier protein. J. Am. Chem. Soc 123, 3385–3386. 10.1021/ja0100827 11457082

[B107] Lamture G. Crooks P. A. Borrelli M. J. (2018). Actinomycin-D and dimethylamino-parthenolide synergism in treating human pancreatic cancer cells. Drug Dev. Res. 79, 287–294. 10.1002/ddr.21441 30295945 PMC6193836

[B108] Lang F. Qu J. Yin H. Li L. Zhi Y. Liu Y. (2018). Apoptotic cell death induced by Z-Ligustilidein human ovarian cancer cells and role of NRF2. Food Chem. Toxicol. 121, 631–638. 10.1016/j.fct.2018.09.041 30243965

[B109] Lascano S. Lopez M. Arimondo P. B. (2018). Natural Products and Chemical Biology Tools: Alternatives to Target Epigenetic Mechanisms in Cancers. Chem. Record 18, 1854–1876. 10.1002/tcr.201800133 30537358

[B110] Lea M. A. Rasheed M. Randolph V. M. Khan F. Shareef A. desBordes C. (2002). Induction of histone acetylation and inhibition of growth of mouse erythroleukemia cells by S-allylmercaptocysteine. Nutr. Cancer 43, 90–102. 10.1207/S15327914NC431_11 12467140

[B111] Lee G. Wolff E. Miller J. H. (2004). Mutagenicity of the cytidine analog zebularine in Escherichia coli. DNA Repair (Amst.) 3, 155–161. 10.1016/j.dnarep.2003.10.010 14706349

[B112] Lee W. J. Shim J.-Y. Zhu B. T. (2005). Mechanisms for the inhibition of DNA methyltransferases by tea catechins and bioflavonoids. Mol. Pharmacol. 68, 1018–1030. 10.1124/mol.104.008367 16037419

[B113] Lee S. J. Krauthauser C. Maduskuie V. Fawcett P. T. Olson J. M. Rajasekaran S. A. (2011). Curcumin-induced HDAC inhibition and attenuation of medulloblastoma growth in vitro and in vivo. BMC Cancer 11, 1–13. 10.1186/1471-2407-11-144 21501498 PMC3090367

[B114] Lee H. S. Park S. B. Kim S. A. Kwon S. K. Cha H. Lee D. Y. (2017). A novel HDAC inhibitor, CG200745, inhibits pancreatic cancer cell growth and overcomes gemcitabine resistance. Sci. Rep. 7, 41615. 10.1038/srep41615 28134290 PMC5278546

[B115] Lewinska A. Adamczyk-Grochala J. Deregowska A. Wnuk M. (2017). Sulforaphane-Induced Cell Cycle Arrest and Senescence are accompanied by DNA Hypomethylation and Changes in microRNA Profile in Breast Cancer Cells. Theranostics 7, 3461–3477. 10.7150/thno.20657 28912888 PMC5596436

[B116] Li Y. Sarkar F. H. (2002). Inhibition of Nuclear Factor κB Activation in PC3 Cells by Genistein Is Mediated via Akt Signaling Pathway. Clin. Cancer Res. 8, 2369–2377. 12114442

[B117] Li Y. Seto E. (2016). HDACs and HDAC Inhibitors in Cancer Development and Therapy. Cold Spring Harb. Perspect. Med. 6, a026831. 10.1101/cshperspect.a026831 27599530 PMC5046688

[B118] Li Y. Zhao T. Liu B. Halaweish I. Mazitschek R. Duan X. (2015). Inhibition of histone deacetylase 6 improves long-term survival in a lethal septic model. J. Trauma Acute Care Surg. 78, 378–385. 10.1097/TA.0000000000000510 25757125 PMC4357277

[B119] Liesch J. M. Sweeley C. C. Staffeld G. D. Anderson M. S. Weber D. J. Scheffer R. P. (1982). Structure of HC-toxin, a cyclic tetrapeptide from helminthosporium carbonum. Tetrahedron 38, 45–48. 10.1016/0040-4020(82)85043-6

[B120] Link A. Balaguer F. Shen Y. Lozano J. J. Leung H.-C. E. Boland C. R. (2013). Curcumin modulates DNA methylation in colorectal cancer cells. PloS One 8, e57709. 10.1371/journal.pone.0057709 23460897 PMC3584082

[B121] Liu Z. Liu S. Xie Z. Pavlovicz R. E. Wu J. Chen P. (2009a). Modulation of DNA methylation by a sesquiterpene lactone parthenolide. J. Pharmacol. Exp. Ther. 329, 505–514. 10.1124/jpet.108.147934 19201992 PMC2672871

[B122] Liu Z. Xie Z. Jones W. Pavlovicz R. E. Liu S. Yu J. (2009b). Curcumin is a potent DNA hypomethylation agent. Bioorg. Med. Chem. Lett. 19, 706–709. 10.1016/j.bmcl.2008.12.041 19112019

[B123] Liu Y. Salvador L. A. Byeon S. Ying Y. Kwan J. C. Law B. K. (2010). Anticolon cancer activity of largazole, a marine-derived tunable histone deacetylase inhibitor. J. Pharmacol. Exp. Ther. 335, 351–361. 10.1124/jpet.110.172387 20739454 PMC2967399

[B124] Ma H. Li L. Dou G. Wang C. Li J. He H. (2017). Z-ligustilide restores tamoxifen sensitivity of ERa negative breast cancer cells by reversing MTA1/IFI16/HDACs complex mediated epigenetic repression of ERa. Oncotarget 8, 29328–29345. 10.18632/oncotarget.16440 28415616 PMC5438733

[B125] Maheshwari R. K. Singh A. K. Gaddipati J. Srimal R. C. (2006). Multiple biological activities of curcumin: a short review. Life Sci. 78, 2081–2087. 10.1016/j.lfs.2005.12.007 16413584

[B126] Majid S. Dar A. A. Ahmad A. E. Hirata H. Kawakami K. Shahryari V. (2009). BTG3 tumor suppressor gene promoter demethylation, histone modification and cell cycle arrest by genistein in renal cancer. Carcinogenesis 30, 662–670. 10.1093/carcin/bgp042 19221000 PMC2664457

[B127] Masuoka Y. Nagai A. Shin-Ya K. Furihata K. Nagai K. Suzuki K.-I. (2001). Spiruchostatins A and B, novel gene expression-enhancing substances produced by Pseudomonas sp. Tetrahedron Lett. 42, 41–44. 10.1016/S0040-4039(00)01874-8

[B128] Matsumoto M. Matsutani S. Sugita K. Yoshida H. Hayashi F. Terui Y. (1992). Depudecin: a novel compound inducing the flat phenotype of NIH3T3 cells doubly transformed by ras- and src-oncogene, produced by Alternaria brassicicola. J. Antibiot. 45, 879–885. 10.7164/antibiotics.45.879 1500354

[B129] Maulucci N. Chini M. G. Di Micco S. Izzo I. Cafaro E. Russo A. (2007). Molecular insights into azumamide e histone deacetylases inhibitory activity. Adv. Ceram. Mater. 129, 3007–3012. 10.1021/ja0686256 17311384

[B130] Mazzone R. Zwergel C. Mai A. Valente S. (2017). Epi-drugs in combination with immunotherapy: a new avenue to improve anticancer efficacy. Clin. Epigenet. 9, 59. 10.1186/s13148-017-0358-y PMC5450222 28572863

[B131] McKinsey T. A. (2012). Therapeutic potential for HDAC inhibitors in the heart. Annu. Rev. Pharmacol. Toxicol. 52, 303–319. 10.1146/annurev-pharmtox-010611-134712 21942627

[B132] Medina-Franco J. L. Caulfield T. (2011). Advances in the computational development of DNA methyltransferase inhibitors. Drug Discovery Today 16, 418–425. 10.1016/j.drudis.2011.02.003 21315180

[B133] Medina-Franco J. L. Yoo J. (2013). Docking of a novel DNA methyltransferase inhibitor identified from high-throughput screening: insights to unveil inhibitors in chemical databases. Mol. Divers. 17, 337–344. 10.1007/s11030-013-9428-z 23447100

[B134] Medina-Franco J. L. López-Vallejo F. Kuck D. Lyko F. (2011). Natural products as DNA methyltransferase inhibitors: a computer-aided discovery approach. Mol. Divers. 15, 293–304. 10.1007/s11030-010-9262-5 20697809

[B135] Medina-Franco J. L. Yoo J. Dueñas-González A. (2015). “Chapter 13 - DNA Methyltransferase Inhibitors for Cancer Therapy,” in Epigenetic technological applications. Ed. Zheng Y.-P. G. (Amsterdam: Academic Press), 265–290.

[B136] Meeran S. M. Patel S. N. Chan T.-H. Tollefsbol T. O. (2011). A novel prodrug of epigallocatechin-3-gallate: differential epigenetic hTERT repression in human breast cancer cells. Cancer Prev. Res. (Phila) 4, 1243–1254. 10.1158/1940-6207.CAPR-11-0009 21411498 PMC3128170

[B137] Mehndiratta S. Lin M.-H. Wu Y.-W. Chen C.-H. Wu T.-Y. Chuang K.-H. (2020). N-alkyl-hydroxybenzoyl anilide hydroxamates as dual inhibitors of HDAC and HSP90, downregulating IFN-γ induced PD-L1 expression. Eur. J. Med. Chem. 185, 111725. 10.1016/j.ejmech.2019.111725 31655430

[B138] Millard C. J. Watson P. J. Celardo I. Gordiyenko Y. Cowley S. M. Robinson C. V. (2013). Class I HDACs share a common mechanism of regulation by inositol phosphates. Mol. Cell 51, 57–67. 10.1016/j.molcel.2013.05.020 23791785 PMC3710971

[B139] Miller A. A. Kurschel E. Osieka R. Schmidt C. G. (1987). Clinical pharmacology of sodium butyrate in patients with acute leukemia. Eur. J. Cancer Clin. Oncol. 23, 1283–1287. 10.1016/0277-5379(87)90109-X 3678322

[B140] Mirza S. Sharma G. Parshad R. Gupta S. D. Pandya P. Ralhan R. (2013). Expression of DNA methyltransferases in breast cancer patients and to analyze the effect of natural compounds on DNA methyltransferases and associated proteins. J. Breast Cancer 16, 23–31. 10.4048/jbc.2013.16.1.23 23593078 PMC3625766

[B141] Mori H. URANO Y. Kinoshita T. Yoshimura S. TAKASE S. HINO M. (2003). FR235222, a fungal metabolite, is a novel immunosuppressant that inhibits mammalian histone deacetylase III. Structure determination. J. Antibiot. 56, 181–185. 10.7164/antibiotics.56.181 12715880

[B142] Mukund V. Mukund D. Sharma V. Mannarapu M. Alam A. (2017). Genistein: Its role in metabolic diseases and cancer. Crit. Rev. Oncol. Hematol. 119, 13–22. 10.1016/j.critrevonc.2017.09.004 29065980

[B143] Murakami T. Sato A. Chun N. A. L. Hara M. Naito Y. Kobayashi Y. (2008). Transcriptional modulation using HDACi depsipeptide promotes immune cell-mediated tumor destruction of murine B16 melanoma. J. Invest. Dermatol. 128, 1506–1516. 10.1038/sj.jid.5701216 18185535

[B144] Musso L. Dallavalle S. Zunino F. (2015). Perspectives in the development of hybrid bifunctional antitumour agents. Biochem. Pharmacol. 96, 297–305. 10.1016/j.bcp.2015.06.006 26074269

[B145] Myzak M. C. Karplus P. A. Chung F.-L. Dashwood R. H. (2004). A novel mechanism of chemoprotection by sulforaphane: inhibition of histone deacetylase. Cancer Res. 64, 5767–5774. 10.1158/0008-5472.CAN-04-1326 15313918

[B146] Myzak M. C. Tong P. Dashwood W.-M. Dashwood R. H. Ho E. (2007). Sulforaphane retards the growth of human PC-3 xenografts and inhibits HDAC activity in human subjects. Exp. Biol. Med. (Maywood) 232, 227–234. 17259330 PMC2267876

[B147] Nair H. K. Rao K. V. K. Aalinkeel R. Mahajan S. Chawda R. Schwartz S. A. (2004). Inhibition of prostate cancer cell colony formation by the flavonoid quercetin correlates with modulation of specific regulatory genes. Clin. Diagn. Lab. Immunol. 11, 63–69. 10.1128/CDLI.11.1.63-69.2004 14715546 PMC321331

[B148] Nakamae S. Toba Y. Takayama K. Sakurai F. Mizuguchi H. (2018). Nanaomycin A Treatment Promotes Hepatoblast Differentiation from Human iPS Cells. Stem Cells Dev. 27, 405–414. 10.1089/scd.2017.0251 29378471

[B149] Nakao Y. Yoshida S. Matsunaga S. Shindoh N. Terada Y. Nagai K. (2006). Azumamides A-E: histone deacetylase inhibitory cyclic tetrapeptides from the marine sponge Mycale izuensis. Angew. Chem. Int. Ed. 45, 7553–7557. 10.1002/anie.200602047 16981208

[B150] Narita K. Kikuchi T. Watanabe K. Takizawa T. Oguchi T. Kudo K. (2009). Total synthesis of the bicyclic depsipeptide HDAC inhibitors spiruchostatins A and B, 5’’-epi-spiruchostatin B, FK228 (FR901228) and preliminary evaluation of their biological activity. Chemistry 15, 11174–11186. 10.1002/chem.200901552 19760730

[B151] Narita K. Fukui Y. Sano Y. Yamori T. Ito A. Yoshida M. (2013). Total synthesis of bicyclic depsipeptides spiruchostatins C and D and investigation of their histone deacetylase inhibitory and antiproliferative activities. Eur. J. Med. Chem. 60, 295–304. 10.1016/j.ejmech.2012.12.023 23313638

[B152] Nelson K. M. Dahlin J. L. Bisson J. Graham J. Pauli G. F. Walters M. A. (2017). The Essential Medicinal Chemistry of Curcumin. J. Med. Chem 60 (5), 1620–1637. 10.1021/acs.jmedchem.6b00975 28074653 PMC5346970

[B153] Nguyen D. D. Wu C. H. Moree W. J. Lamsa A. Medema M. H. Zhao X. L. (2013). MS/MS networking guided analysis of molecule and gene cluster families. Proc. Natl. Acad. Sci. U.S.A. 110, E2611–E2620. 10.1073/pnas.1303471110 23798442 PMC3710860

[B154] Nihal M. Ahmad N. Mukhtar H. Wood G. S. (2005). Anti-proliferative and proapoptotic effects of (-)-epigallocatechin-3-gallate on human melanoma: possible implications for the chemoprevention of melanoma. Int. J. Cancer 114, 513–521. 10.1002/ijc.20785 15609335

[B155] Okamoto H. Fujioka Y. Takahashi A. Takahashi T. Taniguchi T. Ishikawa Y. (2006). Trichostatin A, an inhibitor of histone deacetylase, inhibits smooth muscle cell proliferation via induction of p21(WAF1). J. Atheroscler. Thromb. 13, 183–191. 10.5551/jat.13.183 16908950

[B156] Okano M. Bell D. W. Haber D. A. Li E. (1999). DNA Methyltransferases Dnmt3a and Dnmt3b Are Essential for De Novo Methylation and Mammalian Development. Cell 99, 247–257. 10.1016/S0092-8674(00)81656-6 10555141

[B157] Okura A. Arakawa H. Oka H. Yoshinari T. Monden Y. (1988). Effect of genistein on topoisomerase activity and on the growth of [VAL 12]Ha-ras-transformed NIH 3T3 cells. Biochem. Biophys. Res. Commun. 157, 183–189. 10.1016/S0006-291X(88)80030-5 2848517

[B158] Omar S. H. Al-Wabel N. A. (2010). Organosulfur compounds and possible mechanism of garlic in cancer. Saudi Pharm. J. 18, 51–58. 10.1016/j.jsps.2009.12.007 23960721 PMC3731019

[B159] Park Y. Liu Y. Hong J. Lee C.-O. Cho H. Kim D.-K. (2003). New bromotyrosine derivatives from an association of two sponges, Jaspis wondoensis and Poecillastra wondoensis. J. Nat. Prod. 66, 1495–1498. 10.1021/np030162j 14640526

[B160] Parker L. P. Taylor D. D. Kesterson J. Metzinger D. S. Gercel-Taylor C. (2009). Modulation of microRNA associated with ovarian cancer cells by genistein. Eur. J. Gynaecol. Oncol. 30, 616–621. 20099489

[B161] Pei S. Guzman M. L. Nasim S. Shi L. Crooks P. A. Jordan C. T. (2009). Analysis of the Anti-Leukemia Mechanism of Parthenolide. Blood 114, 2734. 10.1182/blood.V114.22.2734.2734

[B162] Peng S. Zou L. Liu W. Li Z. Liu W. Hu X. (2017). Hybrid liposomes composed of amphiphilic chitosan and phospholipid: Preparation, stability and bioavailability as a carrier for curcumin. Carbohydr. Polym, 156, 322–332. 10.1016/j.carbpol.2016.09.060 27842829

[B163] Perri F. Longo F. Giuliano M. Sabbatino F. Favia G. Ionna F. (2017). Epigenetic control of gene expression: Potential implications for cancer treatment. Crit. Rev. Oncol. Hematol. 111, 166–172. 10.1016/j.critrevonc.2017.01.020 28259291

[B164] Piña I. C. Gautschi J. T. Wang G.-Y.-S. Sanders M. L. Schmitz F. J. France D. (2003). Psammaplins from the sponge Pseudoceratina purpurea: inhibition of both histone deacetylase and DNA methyltransferase. J. Org. Chem. 68, 3866–3873. 10.1021/jo034248t 12737565

[B165] Porter N. J. Christianson D. W. (2017). Binding of the Microbial Cyclic Tetrapeptide Trapoxin A to the Class I Histone Deacetylase HDAC8. ACS Chem. Biol. 12, 2281–2286. 10.1021/acschembio.7b00330 28846375 PMC5600712

[B166] Prieto-Martínez F. D. Peña-Castillo A. Méndez-Lucio O. Fernández-de Gortari E. Medina-Franco J. L. (2016). “Chapter One - Molecular Modeling and Chemoinformatics to Advance the Development of Modulators of Epigenetic Targets: A Focus on DNA Methyltransferases,” in Advances in Protein Chemistry and Structural Biology : Insights into Enzyme Mechanisms and Functions from Experimental and Computational Methods. Ed. Christov C. Z. (Academic Press), 1–26. 10.1016/bs.apcsb.2016.05.001 27567482

[B167] Prince H. M. Dickinson M. Khot A. (2013). Romidepsin for cutaneous T-cell lymphoma. Future Oncol. 9, 1819–1827. 10.2217/fon.13.220 24295412

[B168] Qi M. Xiong X. (2018). Promoter hypermethylation of RARβ2, DAPK, hMLH1, p14, and p15 is associated with progression of breast cancer: A PRISMA-compliant meta-analysis. Med. (Baltimore) 97, e13666. 10.1097/MD.0000000000013666 PMC6320171 30572486

[B169] Qin W. Zhu W. Sauter E. (2005). Resveratrol induced DNA methylation in ER+ breast cancer. Cancer Res. 65, 647.

[B170] Qin W. Zhang K. Clarke K. Weiland T. Sauter E. R. (2014). Methylation and miRNA effects of resveratrol on mammary tumors vs. normal tissue. Nutr. Cancer 66, 270–277. 10.1080/01635581.2014.868910 24447120

[B171] Quiñoà E. Crews P. (1987). Phenolic constituents of. Tetrahedron Lett. 28, 3229–3232. 10.1016/S0040-4039(00)95478-9

[B172] Rajendran P. Kidane A. II Yu T.-W. Dashwood W.-M. Bisson W. H. Löhr C. V. (2013). HDAC turnover, CtIP acetylation and dysregulated DNA damage signaling in colon cancer cells treated with sulforaphane and related dietary isothiocyanates. Epigenetics 8, 612–623. 10.4161/epi.24710 23770684 PMC3857341

[B173] Rehman M. U. Yoshihisa Y. Li P. Zhao L. Q. Narita K. Katoh T. (2014). Spiruchostatin A and B, novel histone deacetylase inhibitors, induce apoptosis through reactive oxygen species-mitochondria pathway in human lymphoma U937 cells. Chem. Biol. Interact. 221, 24–34. 10.1016/j.cbi.2014.07.004 25078973

[B174] Remiszewski S. W. Sambucetti L. C. Atadja P. Bair K. W. Cornell W. D. Green M. A. (2002). Inhibitors of human histone deacetylase: synthesis and enzyme and cellular activity of straight chain hydroxamates. J. Med. Chem. 45, 753–757. 10.1021/jm015568c 11831887

[B175] Remiszewski S. W. (2003). The discovery of NVP-LAQ824: from concept to clinic. Curr. Med. Chem. 10, 2393–2402. 10.2174/0929867033456675 14529481

[B176] Roy M. Sinha D. Mukherjee S. Biswas J. (2011). Curcumin prevents DNA damage and enhances the repair potential in a chronically arsenic-exposed human population in West Bengal, India. Eur. J. Cancer Prev. 20, 123–131. 10.1097/CEJ.0b013e328341017a 21332098

[B177] Roy N. K. Deka A. Bordoloi D. Mishra S. Kumar A. P. Sethi G. (2016). The potential role of boswellic acids in cancer prevention and treatment. Cancer Lett. 377, 74–86. 10.1016/j.canlet.2016.04.017 27091399

[B178] Roy N. K. Parama D. Banik K. Bordoloi D. Devi A. K. Thakur K. K. (2019). An Update on Pharmacological Potential of Boswellic Acids against Chronic Diseases. Int. J. Mol. Sci. 20, 4101. 10.3390/ijms20174101 PMC6747466 31443458

[B179] Royston K. J. Udayakumar N. Lewis K. Tollefsbol T. O. (2017). A Novel Combination of Withaferin A and Sulforaphane Inhibits Epigenetic Machinery, Cellular Viability and Induces Apoptosis of Breast Cancer Cells. Int. J. Mol. Sci. 18, 1092. 10.3390/ijms18051092 PMC5455001 28534825

[B180] Ryu H.-W. Lee D.-H. Shin D.-H. Kim S. H. Kwon S. H. (2015). Aceroside VIII is a new natural selective HDAC6 inhibitor that synergistically enhances the anticancer activity of HDAC inhibitor in HT29 cells. Planta Med. 81, 222–227. 10.1055/s-0034-1396149 25590368

[B181] Saldívar-González F. II Gómez-García A. Chávez-Ponce de León D. E. Sánchez-Cruz N. Ruiz-Rios J. Pilón-Jiménez B. A. (2018). Inhibitors of DNA Methyltransferases From Natural Sources: A Computational Perspective. Front. Pharmacol. 9, 1144. 10.3389/fphar.2018.01144 30364171 PMC6191485

[B182] Sasamura S. SAKAMOTO K. Takagaki S. Yamada T. TAKASE S. MORI H. (2010). AS1387392, a novel immunosuppressive cyclic tetrapeptide compound with inhibitory activity against mammalian histone deacetylase. J. Antibiot. 63, 633–636. 10.1038/ja.2010.51 20588300

[B183] Saunders L. R. Verdin E. (2007). Sirtuins: critical regulators at the crossroads between cancer and aging. Oncogene 26, 5489–5504. 10.1038/sj.onc.1210616 17694089

[B184] Schepper S. Bruwiere H. Verhulst T. Steller U. Andries L. Wouters W. (2003). Inhibition of histone deacetylases by chlamydocin induces apoptosis and proteasome-mediated degradation of survivin. J. Pharmacol. Exp. Ther. 304, 881–888. 10.1124/jpet.102.042903 12538846

[B185] Sekhavat A. Sun J.-M. Davie J. R. (2007). Competitive inhibition of histone deacetylase activity by trichostatin A and butyrate. Biochem. Cell Biol. 85, 751–758. 10.1139/O07-145 18059533

[B186] Shahabipour F. Caraglia M. Majeed M. Derosa G. Maffioli P. Sahebkar A. (2017). Naturally occurring anti-cancer agents targeting EZH2. Cancer Lett. 400, 325–335. 10.1016/j.canlet.2017.03.020 28323035

[B187] Sharma C. Nusri Q. E.-A. Begum S. Javed E. Rizvi T. A. Hussain A. (2012). (-)-Epigallocatechin-3-gallate induces apoptosis and inhibits invasion and migration of human cervical cancer cells. Asian Pac, J. Cancer Prev. 13, 4815–4822. 10.7314/APJCP.2012.13.9.4815 23167425

[B188] Shen S. Kozikowski A. P. (2016). Why Hydroxamates May Not Be the Best Histone Deacetylase Inhibitors–What Some May Have Forgotten or Would Rather Forget? ChemMedChem 11, 15–21. 10.1002/cmdc.201500486 26603496 PMC4765907

[B189] Shen Y. Takahashi M. Byun H.-M. Link A. Sharma N. Balaguer F. (2012). Boswellic acid induces epigenetic alterations by modulating DNA methylation in colorectal cancer cells. Cancer Biol. Ther. 13, 542–552. 10.4161/cbt.19604 22415137 PMC3364790

[B190] Shigematsu N. Ueda H. Takase S. Tanaka H. Yamamoto K. Tada T. (1994). FR901228, a novel antitumor bicyclic depsipeptide produced by Chromobacterium violaceum No. 968. II. 10.7164/antibiotics.47.311 8175483

[B191] Shih Y.-J. Chen Y.-R. Wang K. Whang-Peng J. Tang H.-Y. Lin H.-Y. (2019). “Roles of Resveratrol as Signaling Sensor and Gatekeeper,” in Resveratrol: State-of-the-art science and health applications : actionable targets and mechanisms of resveratrol. Eds. Wu J. M. Hsieh T.-C. (New Jersey: WORLD SCIENTIFIC), 115–144.

[B192] Shindoh N. Mori M. Terada Y. Oda K. Amino N. Kita A. (2008). YM753, a novel histone deacetylase inhibitor, exhibits antitumor activity with selective, sustained accumulation of acetylated histones in tumors in the WiDr xenograft model. Int. J. Oncol 32 (3), 545–555. 10.3892/ijo.32.3.545 [Epub ahead of print]. 18292931

[B193] Shute R. E. Dunlap B. Rich D. H. (1987). Analogues of the cytostatic and antimitogenic agents chlamydocin and HC-toxin: synthesis and biological activity of chloromethyl ketone and diazomethyl ketone functionalized cyclic tetrapeptides. J. Med. Chem. 30, 71–78. 10.1021/jm00384a013 3806605

[B194] Siedlecki P. Boy R. G. Comagic S. Schirrmacher R. Wiessler M. Zielenkiewicz P. (2003). Establishment and functional validation of a structural homology model for human DNA methyltransferase 1. Biochem. Biophys. Res. Commun. 306, 558–563. 10.1016/S0006-291X(03)01000-3 12804601

[B195] Singh S. B. Zink D. L. Polishook J. D. Dombrowski A. W. Darkin-Rattray S. J. Schmatz D. M. (1996). Apicidins: Novel cyclic tetrapeptides as coccidiostats and antimalarial agents from Fusarium pallidoroseum. Tetrahedron Lett. 37, 8077–8080. 10.1016/0040-4039(96)01844-8

[B196] Singh S. B. Zink D. L. Liesch J. M. Dombrowski A. W. Rkin-Rattray S. J. Schmatz D. M. (2001). Structure, histone deacetylase, and antiprotozoal activities of apicidins B and C, congeners of apicidin with proline and valine substitutions. Org. Lett. 3, 2815–2818. 10.1021/ol016240g 11529764

[B197] Singh S. B. Zink D. L. Liesch J. M. Mosley R. T. Dombrowski A. W. Bills G. F. (2002). Structure and chemistry of apicidins, a class of novel cyclic tetrapeptides without a terminal alpha-keto epoxide as inhibitors of histone deacetylase with potent antiprotozoal activities. J. Org. Chem. 67, 815–825. 10.1021/jo016088w 11856024

[B198] Sinha D. Sarkar N. Biswas J. Bishayee A. (2016). Resveratrol for breast cancer prevention and therapy: Preclinical evidence and molecular mechanisms. Semin. Cancer Biol. 40-41, 209–232. 10.1016/j.semcancer.2015.11.001 26774195

[B199] Slingerland M. Guchelaar H.-J. Gelderblom H. (2014). Histone deacetylase inhibitors: an overview of the clinical studies in solid tumors. Anticancer Drugs 25, 140–149. 10.1097/CAD.0000000000000040 24185382

[B200] Soflaei S. S. Momtazi-Borojeni A. A. Majeed M. Derosa G. Maffioli P. Sahebkar A. (2018). Curcumin: A Natural Pan-HDAC Inhibitor in Cancer. Curr. Pharm. Des, 24, 123–129. 10.2174/1381612823666171114165051 29141538

[B201] Soleimani V. Sahebkar A. Hosseinzadeh H. (2018). Turmeric (Curcuma longa) and its major constituent (curcumin) as nontoxic and safe substances: Review. Phytother. Res. 32, 985–995. 10.1002/ptr.6054 29480523

[B202] Son I. H. Lee S. II Yang H. D. Moon H.-I. (2007). Bis(4-hydroxybenzyl)sulfide: a sulfur compound inhibitor of histone deacetylase isolated from root extract of Pleuropterus ciliinervis. Molecules 12, 815–820. 10.3390/12040815 17851433 PMC6149480

[B203] Song J. Noh J. H. Lee J. H. Eun J. W. Ahn Y. M. Kim S. Y. (2005). Increased expression of histone deacetylase 2 is found in human gastric cancer. APMIS 113, 264–268. 10.1111/j.1600-0463.2005.apm_04.x 15865607

[B204] Song J. Teplova M. Ishibe-Murakami S. Patel D. J. (2012). Structure-based mechanistic insights into DNMT1-mediated maintenance DNA methylation. Science 335, 709–712. 10.1126/science.1214453 22323818 PMC4693633

[B205] Souto J. A. Vaz E. Lepore I. Pöppler A.-C. Franci G. Alvarez R. (2010). Synthesis and biological characterization of the histone deacetylase inhibitor largazole and C7- modified analogues. J. Med. Chem. 53, 4654–4667. 10.1021/jm100244y 20491440

[B206] Srivastava R. K. Chen Q. Siddiqui I. Sarva K. Shankar S. (2007). Linkage of curcumin-induced cell cycle arrest and apoptosis by cyclin-dependent kinase inhibitor p21(/WAF1/CIP1). Cell Cycle 6, 2953–2961. 10.4161/cc.6.23.4951 18156803

[B207] Stähelin H. Trippmacher A. (1974). Cytostatic activity of chlamydocin, a rapidly inactivated cyclic tetrapeptide. Eur. J. Cancer (1965) 10, 801–808. 10.1016/0014-2964(74)90137-6 4218812

[B208] Steliou K. Boosalis M. S. Perrine S. P. Sangerman J. Faller D. V. (2012). Butyrate histone deacetylase inhibitors. Biore., Open Access 1, 192–198. 10.1089/biores.2012.0223 PMC3559235 23514803

[B209] Su Z.-Y. Khor T. O. Shu L. Lee J. H. Saw C. L.-L. Wu T.-Y. (2013). Epigenetic reactivation of Nrf2 in murine prostate cancer TRAMP C1 cells by natural phytochemicals Z-ligustilide and Radix angelica sinensis via promoter CpG demethylation. Chem. Res. Toxicol. 26, 477–485. 10.1021/tx300524p 23441843

[B210] Sundaram M. K. Ansari M. Z. Al Mutery A. Ashraf M. Nasab R. Rai S. (2018). Genistein Induces Alterations of Epigenetic Modulatory Signatures in Human Cervical Cancer Cells. Anti-Cancer Agents Med. Chem. 18, 412–421. 10.2174/1871520617666170918142114 28925878

[B211] Suraweera A. O’Byrne K. J. Richard D. J. (2018). Combination Therapy With Histone Deacetylase Inhibitors (HDACi) for the Treatment of Cancer: Achieving the Full Therapeutic Potential of HDACi. Front. Oncol. 8, 92. 10.3389/fonc.2018.00092 29651407 PMC5884928

[B212] Tabudravu J. N. Eijsink V. G. H. Gooday G. W. Jaspars M. Komander D. Legg M. (2002). Psammaplin A, a chitinase inhibitor isolated from the fijian marine sponge Aplysinella rhax. Bioorg. Med. Chem. 10, 1123–1128. 10.1016/S0968-0896(01)00372-8 11836123

[B213] Takahashi M. Sung B. Shen Y. Hur K. Link A. Boland C. R. (2012). Boswellic acid exerts antitumor effects in colorectal cancer cells by modulating expression of the let-7 and miR-200 microRNA family. Carcinogenesis 33, 2441–2449. 10.1093/carcin/bgs286 22983985 PMC3510738

[B214] Tan S. Wang C. Lu C. Zhao B. Cui Y. Shi X. (2009). Quercetin is able to demethylate the p16INK4a gene promoter. Chemotherapy 55, 6–10. 10.1159/000166383 18974642

[B215] Tanaka H. Koyama Y. Awaya J. Marumo H. Oiwa R. (1975). Nanaomycins, new antibiotics produced by a strain of Streptomyces. I. Taxonomy, isolation, characterization and biological properties. J. Antibiot. 28, 860–867. 10.7164/antibiotics.28.860 1201969

[B216] Taori K. Paul V. J. Luesch H. (2008). Structure and activity of largazole, a potent antiproliferative agent from the Floridian marine cyanobacterium Symploca sp. J. Am. Chem. Soc 130, 1806–1807. 10.1021/ja7110064 18205365

[B217] Taunton J. Hassig C. A. Schreiber S. L. (1996). A mammalian histone deacetylase related to the yeast transcriptional regulator Rpd3p. Science 272, 408–411. 10.1126/science.272.5260.408 8602529

[B218] Tiper I. V. Webb T. J. (2016). Histone deacetylase inhibitors enhance CD1d-dependent NKT cell responses to lymphoma. Cancer Immunol. Immunother. 65, 1411–1421. 10.1007/s00262-016-1900-z 27614429 PMC5580026

[B219] Toden S. Okugawa Y. Buhrmann C. Nattamai D. Anguiano E. Baldwin N. (2015). Novel Evidence for Curcumin and Boswellic Acid-Induced Chemoprevention through Regulation of miR-34a and miR-27a in Colorectal Cancer. Cancer Prev. Res. (Phila) 8, 431–443. 10.1158/1940-6207.CAPR-14-0354 25712055 PMC4417447

[B220] Traisaeng S. Herr D. R. Kao H.-J. Chuang T.-H. Huang C.-M. (2019). A Derivative of Butyric Acid, the Fermentation Metabolite of Staphylococcus epidermidis, Inhibits the Growth of a Staphylococcus aureus Strain Isolated from Atopic Dermatitis Patients. Toxins (Basel) 11, 311. 10.3390/toxins11060311. PMC6628397 31159213

[B221] Tsuji N. Kobayashi M. Nagashima K. Wakisaka Y. Koizumi K. (1976). A new antifungal antibiotic, trichostatin. J. Antibiot. 29, 1–6. 10.7164/antibiotics.29.1 931784

[B222] Ueda H. Nakajima H. Hori Y. Fujita T. Nishimura M. Goto T. (1994). FR901228, a novel antitumor bicyclic depsipeptide produced by Chromobacterium violaceum No. 968. I. 10.7164/antibiotics.47.301 7513682

[B223] Uramova S. Kubatka P. Dankova Z. Kapinova A. Zolakova B. Samec M. (2018). Plant natural modulators in breast cancer prevention: status quo and future perspectives reinforced by predictive, preventive, and personalized medical approach. EPMA J. 9, 403–419. 10.1007/s13167-018-0154-6 30538792 PMC6261910

[B224] van den Eynde M. D. G. Geleijnse J. M. Scheijen J. L. J. M. Hanssen N. M. J. Dower J. II Afman L. A. (2018). Quercetin, but Not Epicatechin, Decreases Plasma Concentrations of Methylglyoxal in Adults in a Randomized, Double-Blind, Placebo-Controlled, Crossover Trial with Pure Flavonoids. J. Nutr. 148, 1911–1916. 10.1093/jn/nxy236 30398646

[B225] Vandermolen K. M. McCulloch W. Pearce C. J. Oberlies N. H. (2011). Romidepsin (Istodax, NSC 630176, FR901228, FK228, depsipeptide): a natural product recently approved for cutaneous T-cell lymphoma. J. Antibiot. 64, 525–531. 10.1038/ja.2011.35 21587264 PMC3163831

[B226] Vijayaraghavalu S. Dermawan J. K. Cheriyath V. Labhasetwar V. (2013). Highly synergistic effect of sequential treatment with epigenetic and anticancer drugs to overcome drug resistance in breast cancer cells is mediated via activation of p21 gene expression leading to G2/M cycle arrest. Mol. Pharm. 10, 337–352. 10.1021/mp3004622 23215027 PMC3540132

[B227] Villadsen J. S. Stephansen H. M. Maolanon A. R. Harris P. Olsen C. A. (2013). Total synthesis and full histone deacetylase inhibitory profiling of Azumamides A-E as well as β²- epi-Azumamide E and β³-epi-Azumamide E. J. Med. Chem. 56, 6512–6520. 10.1021/jm4008449 23865683

[B228] Walton J. D. Earle E. D. (1983). The epoxide in HC-toxin is required for activity against susceptible maize. Physiol. Plant Pathol. 22, 371–376. 10.1016/S0048-4059(83)81025-X

[B229] Walton J. D. Earle E. D. Stähelin H. Grieder A. Hirota A. Suzuki A. (1985). Reciprocal biological activities of the cyclic tetrapeptides chlamydocin and HC-toxin. Experientia 41, 348–350. 10.1007/BF02004498 3918884

[B230] Walton L. J. Corre C. Challis G. L. (2006). Mechanisms for incorporation of glycerol-derived precursors into polyketide metabolites. J. Ind. Microbiol. Biotechnol. 33, 105–120. 10.1007/s10295-005-0026-7 16187096

[B231] Wang S.-C. Lee T.-H. Hsu C.-H. Chang Y.-J. Chang M.-S. Wang Y.-C. (2014). Antroquinonol D, isolated from Antrodia camphorata, with DNA demethylation and anticancer potential. J. Agric. Food Chem. 62, 5625–5635. 10.1021/jf4056924 24784321

[B232] Wang W. He Y. Yu G. Li B. Sexton D. W. Wileman T. (2015). Sulforaphane Protects the Liver against CdSe Quantum Dot-Induced Cytotoxicity. PloS One 10, e0138771. 10.1371/journal.pone.0138771 26402917 PMC4581733

[B233] Wei B.-L. Chen Y.-C. Hsu H.-Y. (2011). Kazinol Q from Broussonetia kazinoki enhances cell death induced by Cu(II) through increased reactive oxygen species. Molecules 16, 3212–3221. 10.3390/molecules16043212 21499221 PMC6260624

[B234] Wen S. Carey K. L. Nakao Y. Fusetani N. Packham G. Ganesan A. (2007). Total synthesis of azumamide A and azumamide E, evaluation as histone deacetylase inhibitors, and design of a more potent analogue. Org. Lett. 9, 1105–1108. 10.1021/ol070046y 17311393

[B235] Wen J. Bao Y. Niu Q. Liu J. Yang J. Wang W. (2016). Synthesis, biological evaluation and molecular modeling studies of psammaplin A and its analogs as potent histone deacetylases inhibitors and cytotoxic agents. Bioorg. Med. Chem. Lett. 26, 4372–4376. 10.1016/j.bmcl.2015.12.094 27460171

[B236] Weng J.-R. Lai I.-L. Yang H.-C. Lin C.-N. Bai L.-Y. (2014). Identification of kazinol Q, a natural product from Formosan plants, as an inhibitor of DNA methyltransferase. Phytother. Res. 28, 49–54. 10.1002/ptr.4955 23447335

[B237] Wilcox R. A. (2016). Cutaneous T-cell lymphoma: 2016 update on diagnosis, risk-stratification, and management. Am. J. Hematol. 91, 151–165. 10.1002/ajh.24233 26607183 PMC4715621

[B238] Xie Q. Bai Q. Zou L.-Y. Zhang Q.-Y. Zhou Y. Chang H. (2014). Genistein inhibits DNA methylation and increases expression of tumor suppressor genes in human breast cancer cells. Genes Chromosomes Cancer 53, 422–431. 10.1002/gcc.22154 24532317

[B239] Yang C. S. Wang X. (2010). Green tea and cancer prevention. Nutr. Cancer 62, 931–937. 10.1080/01635581.2010.509536 20924968

[B240] Ying Y. Taori K. Kim H. Hong J. Luesch H. (2008). Total synthesis and molecular target of largazole, a histone deacetylase inhibitor. J. Am. Chem. Soc. 130, 8455–8459. 10.1021/ja8013727 18507379

[B241] Yoo J. Medina-Franco J. L. (2011). Homology modeling, docking and structure-based pharmacophore of inhibitors of DNA methyltransferase. J. Comput. Aided Mol. Des. 25, 555–567. 10.1007/s10822-011-9441-1 21660514

[B242] Yoshida M. Kijima M. Akita M. Beppu T. (1990). Potent and specific inhibition of mammalian histone deacetylase both in vivo and in vitro by trichostatin A. J. Biol. Chem. 265, 17174–17179. 2211619

[B243] Yoshida M. Horinouchi S. Beppu T. (1995). Trichostatin A and trapoxin: novel chemical probes for the role of histone acetylation in chromatin structure and function. Bioessays 17, 423–430. 10.1002/bies.950170510 7786288

[B244] Yu J. Peng Y. Wu L.-C. Xie Z. Deng Y. Hughes T. (2013). Curcumin down-regulates DNA methyltransferase 1 and plays an anti-leukemic role in acute myeloid leukemia. PloS One 8, e55934. 10.1371/journal.pone.0055934 23457487 PMC3572185

[B245] Zhang Y. Kensler T. W. Cho C. G. Posner G. H. Talalay P. (1994). Anticarcinogenic activities of sulforaphane and structurally related synthetic norbornyl isothiocyanates. Proc. Natl. Acad. Sci. U.S.A. 91, 3147–3150. 10.1073/pnas.91.8.3147 8159717 PMC43532

[B246] Zhang F. Yang Y. Su P. Guo Z. (2009). Microwave-assisted extraction of rutin and quercetin from the stalks of Euonymus alatus (Thunb.) Sieb. Phytochem. Anal. 20, 33–37. 10.1002/pca.1088 19086093

[B247] Zhang Y. Zhou L. Bao Y. L. Wu Y. Yu C. L. Huang Y. X. (2010). Butyrate induces cell apoptosis through activation of JNK MAP kinase pathway in human colon cancer RKO cells. Chem. Biol. Interact. 185, 174–181. 10.1016/j.cbi.2010.03.035 20346929

[B248] Zhang H. Wang K. Lin G. Zhao Z. (2014). Antitumor mechanisms of S-allyl mercaptocysteine for breast cancer therapy. BMC Complement. Altern. Med. 14, 270. 10.1186/1472-6882-14-270 25070343 PMC4122773

[B249] Zhao J. Huang W.-G. He J. Tan H. Liao Q.-J. Su Q. (2006). Diallyl disulfide suppresses growth of HL-60 cell through increasing histone acetylation and p21WAF1 expression in vivo and in vitro. Acta Pharmacol. Sin. 27, 1459–1466. 10.1111/j.1745-7254.2006.00433.x 17049122

[B250] Zhao F. Zhang J. Chang Na. (2018). Epigenetic modification of Nrf2 by sulforaphane increases the antioxidative and anti-inflammatory capacity in a cellular model of Alzheimer’s disease. Eur. J. Pharmacol. 824, 1–10. 10.1016/j.ejphar.2018.01.046 29382536

[B251] Zhou D.-H. Wang X. Yang M. Shi X. Huang W. Feng Q. (2013). Combination of low concentration of (-)-epigallocatechin gallate (EGCG) and curcumin strongly suppresses the growth of non-small cell lung cancer in vitro and in vivo through causing cell cycle arrest. Int. J. Mol. Sci. 14, 12023–12036. 10.3390/ijms140612023 23739680 PMC3709771

[B252] Zhou W. Chen X. He K. Xiao J. Duan X. Huang R. (2016). Histone deacetylase inhibitor screening identifies HC toxin as the most effective in intrahepatic cholangiocarcinoma cells. Oncol. Rep. 35, 2535–2542. 10.3892/or.2016.4636 26935789 PMC4811396

[B253] Zhou Z.-H. Yang J. Kong A.-N. (2017). Phytochemicals in Traditional Chinese Herbal Medicine: Cancer Prevention and Epigenetics Mechanisms. Curr. Pharmacol. Rep. 3, 77–91. 10.1007/s40495-017-0086-1

[B254] Zubieta C. He X. Z. Dixon R. A. Noel J. P. (2001). Structures of two natural product methyltransferases reveal the basis for substrate specificity in plant O-methyltransferases. Nat. Struct. Biol. 8, 271–279. 10.1038/85029 11224575

[B255] Zwergel C. Valente S. Mai A. (2016). DNA Methyltransferases Inhibitors from Natural Sources. Curr. Top. Med. Chem. 16, 680–696. 10.2174/1568026615666150825141505 26303417

